# Recent advances in Alzheimer’s disease: mechanisms, clinical trials and new drug development strategies

**DOI:** 10.1038/s41392-024-01911-3

**Published:** 2024-08-23

**Authors:** Jifa Zhang, Yinglu Zhang, Jiaxing Wang, Yilin Xia, Jiaxian Zhang, Lei Chen

**Affiliations:** 1 Department of Neurology, Laboratory of Neuro-system and Multimorbidity and State Key Laboratory of Biotherapy and Cancer Center, West China Hospital, Sichuan University, Chengdu, 610041 Sichuan China; 2 Department of Pharmaceutical Sciences, College of Pharmacy, University of Tennessee Health Science Center, Memphis, 38163 TN USA

**Keywords:** Medicinal chemistry, Drug discovery

## Abstract

Alzheimer’s disease (AD) stands as the predominant form of dementia, presenting significant and escalating global challenges. Its etiology is intricate and diverse, stemming from a combination of factors such as aging, genetics, and environment. Our current understanding of AD pathologies involves various hypotheses, such as the cholinergic, amyloid, tau protein, inflammatory, oxidative stress, metal ion, glutamate excitotoxicity, microbiota-gut-brain axis, and abnormal autophagy. Nonetheless, unraveling the interplay among these pathological aspects and pinpointing the primary initiators of AD require further elucidation and validation. In the past decades, most clinical drugs have been discontinued due to limited effectiveness or adverse effects. Presently, available drugs primarily offer symptomatic relief and often accompanied by undesirable side effects. However, recent approvals of aducanumab (**1**) and lecanemab (**2**) by the Food and Drug Administration (FDA) present the potential in disrease-modifying effects. Nevertheless, the long-term efficacy and safety of these drugs need further validation. Consequently, the quest for safer and more effective AD drugs persists as a formidable and pressing task. This review discusses the current understanding of AD pathogenesis, advances in diagnostic biomarkers, the latest updates of clinical trials, and emerging technologies for AD drug development. We highlight recent progress in the discovery of selective inhibitors, dual-target inhibitors, allosteric modulators, covalent inhibitors, proteolysis-targeting chimeras (PROTACs), and protein-protein interaction (PPI) modulators. Our goal is to provide insights into the prospective development and clinical application of novel AD drugs.

## Introduction

Dementia has emerged as a global health challenge. According to the World Health Organization’s 2022 blueprint for dementia research, an estimated 55.2 million individuals globally are affected. The prevalence among those over the age of 60 varies by region: with Southeast Asia reporting a prevalence of 2.9%, Europe at 6.5%, and other regions experiencing rates between 3.1% and 5.7%.^1^ The incidence of dementia is generally increasing, while some high-income countries are seeing a decline.^2^ By 2030, the estimated number of people living with dementia will surge to 78 million. Furthermore, the global financial burden associated with medical care, social services, and informal caregiving for those with dementia is expected to exceed US$ 2.8 trillion. This situation will have a profound impact on individuals, families, and societies.^1^ Alzheimer’s disease (AD), the predominant form of dementia, exhibits similar epidemiological trends and represents an urgent and escalating challenge worldwide. In the United States, approximately one in nine individuals (10.8%) age 65 and older suffer from AD, with an annual incidence of 1275 new cases per 100,000 persons.^3,4^ Patients with AD exhibit a substantial accumulation of amyloid-β (Aβ) plaques and neurofibrillary tangles (NFTs) within their brains, accompanied by a cascade of pathological processes such as neuroinflammation, synaptic dysfunction, mitochondrial and bioenergetic disturbances, as well as vascular abnormalities. Collectively these processes may ultimately lead to the death of neurons.^5,6^ Clinically, the primary hallmark of AD is amnestic cognitive impairment. Initially, symptoms may manifest as depression, anxiety, social withdrawal, and altered sleep patterns. As the disease progresses, symptoms worsen, leading to severe memory loss, neuropsychiatric symptoms such as hallucinations and delusions, and intensified behavioral and emotional issues in its advanced stages. Additionally, some patients with non-amnestic cognitive impairment may experience varying levels of dysfunctions in visual-spatial, language, executive functions, behavior, or motor skills.^2,7–9^ Moreover, comorbidities linked with AD may exacerbate the health condition of patients, contributing to clinical phenotype diversity and accelerating cognitive dysfunction. Such conditions include hypercholesterolemia, hypertension, diabetes, obesity, depression, and cardiovascular diseases. Additionally, complications arising from AD progressions, like thrombosis, mobility impairments, dysphagia, malnutrition, and pneumonia (lung infections), may considerably diminish the life quality of patients and increase mortality risk.^2,4,10–14^ The connection between comorbidities and the pathological changes in AD is currently the subject of ongoing research.^15–17^ Unfortunately, there is yet no cure for AD, and patients are frequently diagnosed at a late and irreversible stage, facing an average survival period of 4–8 years.^4,18,19^ Nonetheless, pathological changes in the brain begin during the preclinical stage, decades before clinical symptoms. Typically, patients transit to mild cognitive impairment (MCI) around 6-10 years later, with approximately 15% progressing to AD within 2 years and one-third within 5 years.^4,20,21^ Therefore, it’s crucial to concentrate on the preclinical and MCI stages, where early intervention and management of modifiable risk factors could potentially lower the risk of onset or delay the progression of disease.^22^ Evidence suggests that about one-third of AD cases worldwide are closely linked to modifiable risk factors.^23^ Encouragingly, due to improvements in risk factors such as vascular health, lifestyle choices, and education levels, the incidence of AD is on a downward trend in the United States, South Korea, Europe, and certain regions of Asia.^2,24^ In recent years, numerous articles^4,22,23,25–28^ have highlighted modifiable risk factors for AD, alongside the benefits of Multidomain Alzheimer Preventive Trials. These insights underscore the efficacy of early prevention strategies for AD.

**Fig. 1:**
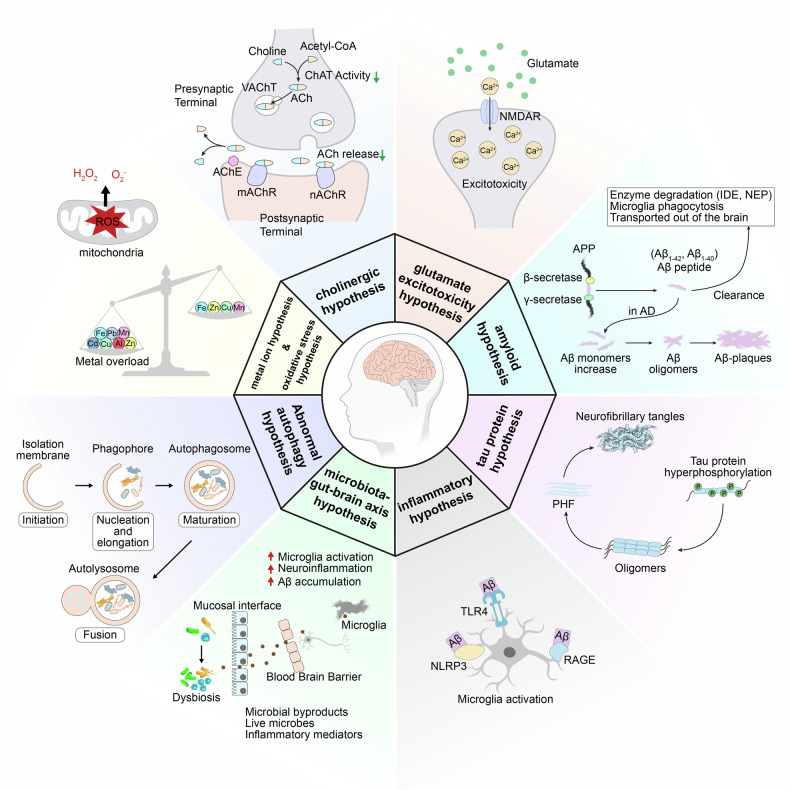
Diagram for the pathogenesis of AD, including the cholinergic hypothesis,^619,620^ the glutamatergic hypothesis,^621^ the amyloid hypothesis,^622,623^ the tau protein hypothesis,^624,625^ the inflammatory hypothesis,^626,627^ the microbiota-gut-brain axis hypothesis,^628,629^ the oxidative stress hypothesis,^191^ the metal ion hypothesis,^630,631^ and the abnormal autophagy hypothesis^235^

The etiology of AD is complex and diverse, and the precise mechanisms underlying its onset are not yet completely understood. Beyond the pivotal role of Aβ and tau, a spectrum of other factors may contribute to the pathology of AD, such as acetylcholine deficiency, neuroinflammation, oxidative stress, biometal dyshomeostasis, glutamate imbalance, insulin resistance, gut microbiome abnormalities, cholesterol homeostasis disruption, mitochondrial dysfunction, and autophagy abnormalities^29–31^ (Fig. 1). Of note, these factors also form the foundation for clinical diagnosis and treatment strategies. Biomarkers can identify patients in the early stages, monitor disease progression, and evaluate the effectiveness of drugs.^32–35^ The hypotheses surrounding these pathogenic factors provide potential targets for drug development. However, the development of effective AD drugs has been fraught with challenges. Tacrine (**3**)^36–40^ was withdrawn from the market primarily because of its hepatotoxicity. Medications such as donepezil (**4**),^41–43^ rivastigmine (**5**),^44,45^ galantamine (**6**),^46–48^ memantine (**7**),^49,50^ and namzaric (**8**)^51,52^ have been employed in clinical settings. While these drugs can temporarily alleviate or stabilize symptoms, they are unable to stop the long-term progression of the disease and are associated with various side effects.^33,53^ New drugs, including sodium oligomannate (**9**, GV-971),^54–56^ aducanumab (**1**),^57–59^ lecanemab (**2**),^60–62^ and donanemab (**10**, currently under review for market approval),^63^ which aim to offer disease-modifying therapies that intervene in the progression of AD. Their clinical relevance remains to be evaluated thoroughly. More than a century has elapsed since AD was first described in 1906,^64^ and significant progress has been made in understanding its pathogenesis, improving diagnosis, and enhancing treatment.^65,66^ Unfortunately, the current offerings fall short of meeting the need to address cognitive. Therefore, this review takes into account the AD research framework of prevention, diagnosis, and treatment, and discusses the pathogenesis, diagnostic biomarkers, clinical trials, and next-generation small molecule drugs. It also emphasizes the critical need to improve the safety and efficacy of drugs through innovative drug development techniques, such as selective inhibitors,^67^ dual-target inhibitors,^68,69^ allosteric modulators,^70,71^ covalent inhibitors,^72^ proteolysis-targeting chimeras (PROTACs)^73^ and protein-protein interaction (PPI) modulators,^74,75^ aiming for more effective clinical translation from outcomes of research.

## Mechanisms of AD

Numerous hypotheses have been proposed to unravel the pathogenesis of AD, yet a unified theory remains elusive, likely due to the complex nature of AD. AD can be categorized into two main types: familial (accounting for 1-5% of AD cases) and sporadic forms (over 95% of cases).^76^ Familial AD (FAD) is predominantly characterized by autosomal dominant genetic mutations in amyloid precursor protein (APP), presenilin 1 (PS1), and presenilin 2 (PS2) genes, typically manifesting between 30-65 years and progressing rapidly. In contrast, sporadic AD (SAD), also known as late-onset AD, usually manifests after the age of 65 and is influenced by a combination of genetic risks, environmental factors, and various comorbidities.^77–79^ Genome-wide association studies (GWAS) and genome-wide meta-analyses have identified numerous genetic risk loci associated with SAD, implicating pathways in immune response, lipid metabolism, Aβ plaque, NFTs, and endocytosis, yet many loci remain undiscovered.^80–83^ Non-genetic factors such as lifestyles, psychosocial factors, environment, and diseases related to AD (comorbidities and complications), may elevate the risk of developing AD. They may achieve this by altering biological pathways and genetic susceptibility,^23,84–86^ making it challenging to pinpoint a direct cause of clinical pathology in AD. Furthermore, different AD subtypes (typical and atypical) often exhibit various clinical symptoms.^87–89^ Thirdly, AD has multiple pathological features including Aβ plaques, NFTs, synaptic and neuronal loss, and neuroinflammation.^90,91^ Overall, the diversity of triggers, clinical manifestations, and neuropathological features underlie the heterogeneity of AD. Consequently, developing a comprehensive theoretical framework that links genetic foundations, molecular mechanisms, and clinical phenotypes of AD is extremely challenging. Current limitations in AD research also hinder our comprehensive understanding of its pathophysiology.^1^ Moreover, the high failure rate of clinical trials makes it difficult to effectively validate hypotheses, possibly attributed to the coexistence of multiple theories (which will be detailed in subsequent sections).

### Cholinergic hypothesis

The cholinergic hypothesis was the earliest to delineate the pathogenesis of AD. It describes the severe damage of cholinergic neurons in the nucleus basalis of meynert (NBM), leading to a marked decrease in choline acetyltransferase (ChAT) activity within the primary projection areas - the cerebral cortex and hippocampus (regions associated with learning and memory). Additionally, this neuronal damage is accompanied by a significant increase in the density of senile plaques. The scenario in the cholinergic hypothesis suggests a close relationship between deficits of basal forebrain cholinergic and cognitive impairments observed in AD.^91–97^ Cholinergic neurons in the basal forebrain are crucial components of the central cholinergic system, significant contributing to the regulation of cognitive functions, attention, and memory.^98^ These cell bodies of neurons are predominantly located in the medial septal nucleus (MSN), diagonal band of broca (DBB), NBM, and substantia innominata (SI).^97,99^ It has been observed that cholinergic neurons in the NBM region are particularly susceptible to degeneration and loss in AD. It is believed to be associated with nerve growth factor (NGF)-dependent nutritional depletion.^100,101^ Acetylcholine (ACh) is synthesized from choline and acetyl-coenzyme A by ChAT, then transported into synaptic vesicles through the vesicular acetylcholine transporter (VAChT). When a neural signal arrives, ACh is released, where it binds to muscarinic and nicotinic acetylcholine receptors (mAChRs and nAChRs) on the postsynaptic membrane to transmit neural signals. Subsequently, ACh in the synaptic cleft is degraded into choline by acetylcholinesterase (AChE) and reabsorbed into presynaptic cholinergic neurons.^31,102–104^ The decline in the activity of ChAT, combined with the detrimental effects of Aβ on nutritional imbalance, the synthesis, release, and degradation of ACh, leads to a reduction of ACh levels. This decrease impairs its physiological functions in learning, memory, motor regulation, and sleep cycle regulation.^97,105–108^ In summary, the cholinergic hypothesis, as a well-established and classic theory, has significantly advanced the early research and drug development for AD. AChE inhibitors (AChEIs), like donepezil (**4**), rivastigmine (**5**), and galantamine (**6**), which are approved over two decades ago, remain the mainstay of AD treatment in clinical management.^109^ Despite these advancements, the limited efficacy and side effects of such drugs, coupled with the presence of non-cholinergic groups in AD,^99^ and non-specificity in these pathological features,^94^ challenge the cholinergic hypothesis to fully explain the complex of AD pathology.

### Amyloid hypothesis

**Fig. 2:**
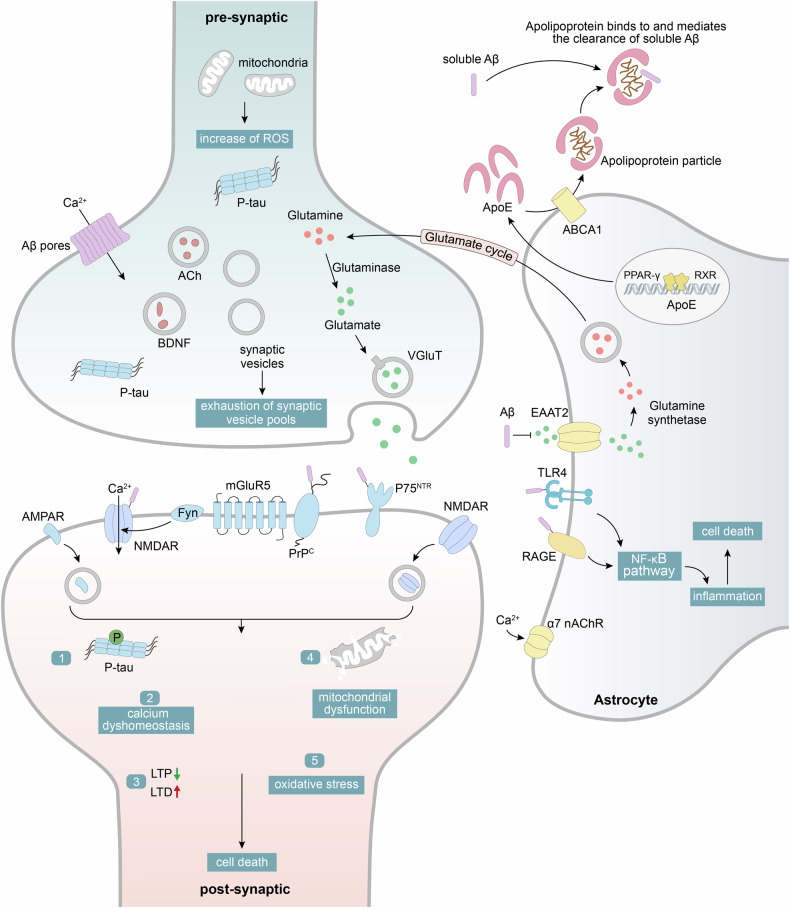
Schematic illustration depicting the possible molecular downstream pathways of Aβ on neuronal synapses and astrocytes. (1) Aβ is capable of interacting with cell membranes and binding to a variety of synaptic receptors such as PrP^C^, NMDA receptors, P75^NTR^, and mGluR5, which leads to a cascade of events including calcium dyshomeostasis, inhibition of long-term potentiation (LTP), tau hyperphosphorylation, mitochondrial dysfunction, and oxidative stress, ultimately resulting in neuronal death.^112,632,633^ (2) Aβ blocks the reuptake of glutamate by excitatory amino acid transporter (EAAT) receptors, causing glutamate accumulation intersynaptically and neuronal hyperactivity.^634^ (3) Aβ and some pro-inflammatory cytokines (such as TNFα, IL-1α, and C1q) may induce the A1 phenotype of astrocytes. This transformation may involve altering astrocyte functions and modulating their interactions with other cells (such as neurons and microglia), thereby participating in processes such as Aβ deposition, neuroinflammation, synaptic loss, and neuronal death.^635–637^ (4) APOE, primarily released from astrocytes, associates with lipoproteins to form APOE-associated lipoprotein particles, which can bind to soluble Aβ and mediate its clearance^119^

**Fig. 3:**
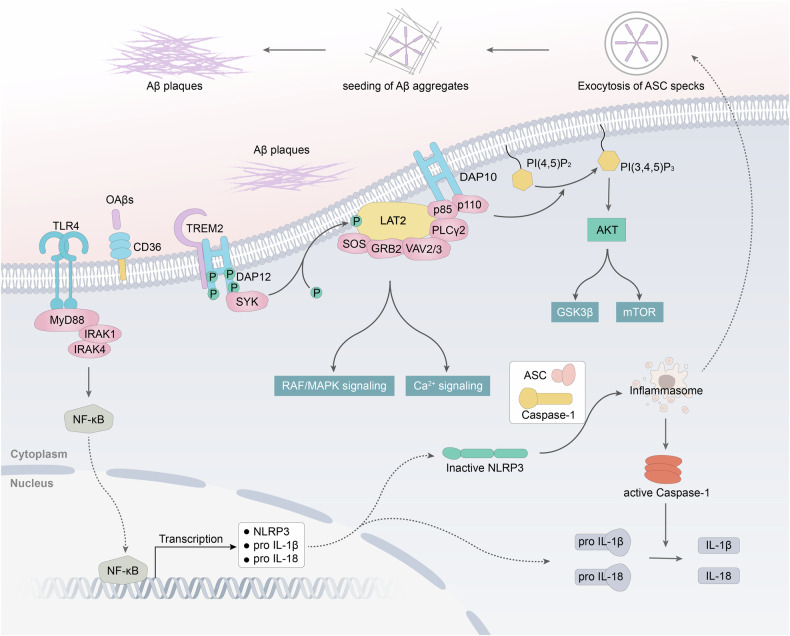
Schematic illustration depicting potential molecular downstream pathways of Aβ on microglia. Microglia has numerous pattern recognition receptors that can bind to Aβ, initiating an inflammatory cascade. This process promotes the assembly and activation of NLRP3, leading to the release of pro-inflammatory cytokines, which further exacerbate the aggregation of Aβ.^171^ In addition, the diagram also encompasses the downstream signaling pathways of TREM2.^638,639^ Some variants associated with AD, such as the TREM2 variant R47H, may potentially diminish the binding or internalization of TREM2 with ligands such as APOE-Aβ complexes, APOE, phospholipids, and Aβ. This reduction may consequently impair the activation of microglial cells, thereby compromising their ability to clear amyloid plaques.^638,640–643^ It is worth noting that there remain many uncertainties and controversies regarding the in vivo ligands and signaling pathways of TREM2, as well as the relationship between TREM2 variants and AD. Future in vivo experiments are needed to elucidate these aspects

The accumulation of Aβ is a hallmark pathological feature in both extensively studied autosomal dominant AD and sporadic late-onset AD patients.^110^ Aβ originates from the processing of the APP, a transmembrane glycoprotein, through its sequential cleavage by β-secretase and γ-secretase (a multiprotein complex with PS1 or PS2 as catalytic subunits). This process yields various lengths of Aβ fragments, with Aβ_40_ and Aβ_42_ being the predominant. The hydrophobic C-terminal of Aβ_42_ facilitates the β-sheet conformational transition and the aggregation and formation of the core component of senile plaques.^78,111,112^ Mutations in PS1, a typical mutation in FAD, potentially promote Aβ accumulation through multiple mechanisms, including increased Aβ production and impairment of autophagy functions.^83,113–115^ However, FAD mutations are not necessarily linked to an increase in Aβ_42_ levels or an elevation of Aβ_42_/Aβ_40_ ratio.^78,116^ The plaque formation in SAD is notably more intricate, related to a dynamic imbalance between Aβ production and clearance mechanisms.^117^ Apolipoprotein E (APOE), particularly the ε4 allele, stands out as the most crucial genetic risk factor for SAD. Carrying one or two APOE ε4 alleles increases the risk of AD by 2-3 and 12-fold, respectively.^118^ Research indicates that APOE protein is detectable in neuritic plaques, and individuals with the APOEε4 allele also have a higher burden of Aβ plaques in their brains,^119,120^ highlighting its critical influences on Aβ deposition. While the exact mechanisms remain to be agreed upon, both in vitro and in vivo experiments suggested several potential pathways for APOEε4, including enhancing Aβ production (promoting APP transcription and processing), facilitating Aβ aggregation (interaction with soluble and fibrillary Aβ aids in seeding/oligomerization/protofibril formation), and impairing Aβ clearance (disrupted glial and enzymatic Aβ degradation functions, and Aβ removal rate from the brain).^121–124^ Moreover, other genetic risk factors,^125,126^ cardiovascular health issues (such as diabetes, hypercholesterolemia), and lifestyle factors (such as diet and sleep)^127^ have also been extensively studied in recent years for their relationship with Aβ metabolism in SAD. The toxicity mechanism of Aβ aggregates remains uncertain, but different perspectives exist:^77,128^ Aβ might cause AD pathology through the loss of physiological functions during the aggregation process. Aβ monomers have neuroprotective properties, with assumed roles in antioxidant and antimicrobial activities, improving the condition of damaged nervous systems, regulating the vascular system, and enhancing synaptic plasticity.^129,130^ Soluble Aβ oligomers are the primary neurotoxic substances,^131–133^ disruption of cell membrane integrity,^134^ activation in inflammatory responses,^135,136^ causes of calcium homeostasis imbalance^137^ and mitochondrial dysfunction,^138–140^ triggers in oxidative stress,^141^ and damage factor of synapses.^142^ The potential downstream pathways of oligomers on neurons and glial cells are illustrated in Fig. 2 and Fig. 3. The amyloid cascade^143^ has been proposed for over 30 years, which provided crucial insights into the mechanisms of AD’s onset and progression. This hypothesis has led to the development of drugs, including β-secretase inhibitors, γ-secretase inhibitors and modulators, anti-amyloid antibodies, Aβ vaccine, and Aβ aggregation inhibitors, aimed at delaying the disease’s advancement. Currently, antibodies like aducanumab (**1**), lecanemab (**2**), and donanemab (**10**) show their promise in proving Aβ as a significant factor in AD development. However, in light of beneficial effects on reducing Aβ brain burden, the clinical value of these drugs remains to be validated.^77,78^ Of note, the amyloid cascade hypothesis remains controversial. This theory faces challenges in explaining the diverse pathological features, shows a weak correlation between Aβ and cognitive decline, and has failed to demonstrate efficacy in numerous clinical drugs to target Aβ.^118,144–147^ These findings suggest that Aβ deposition or plaque formation might not be the actual cause of the disease, but rather a result or secondary factor of the pathological process.^77,148^ Given the increasingly recognized critical role of tau, the pathological sequence and interplay of tau and Aβ in AD deserve further exploration.^149–151^

### Tau protein hypothesis

As a major component of NFTs, tau protein exhibits a spatial and temporal distribution that strongly correlates with clinical symptoms, making it a highly specific pathological biomarker in AD patients.^152^ Tau is a microtubule-associated protein predominantly expressed in the axons of neurons, with lower expression levels in dendrites, soma, and glial cells.^153,154^ It hosts numerous phosphorylation sites across its N-terminal region, C-terminal region, and repeat region, which are regulated by a balance of various kinases and phosphatases to maintain normal neuronal physiological functions.^150,155^ Under pathological conditions, an imbalanced activity of phosphatases and kinases leads to hyperphosphorylation of tau.^156,157^ This process leads to the detachment of tau protein from microtubules, followed by conformational changes and mislocalization, accumulation of tau oligomers, paired helical filaments (PHFs), and NFTs within the cell body and dendrites. These changes ultimately impair neuronal function and cause cell death.^158–160^ Additionally, other post-translational modifications, including truncation,^161,162^ glycosylation,^163^ glycation,^164^ and sumoylation,^165^ play an active role in promoting tau aggregation and increasing its toxicity. Tau oligomers not only generate neurotoxicity within cells but also facilitate pathological spread through synaptic transmission. This process induces the aggregation of monomeric tau in recipient neurons, leading to the formation of new oligomers.^166^ Overall, the significance of tau in AD pathogenesis stems from the strong correlation between tau accumulation and cognitive symptoms.^152^ In recent years, there has been a heightened focus on tau deposition, including the correlation between tau deposition, brain atrophy, and glucose metabolism in both typical and atypical AD,^167,168^ as well as the effects of tau deposition at the molecular and cellular levels.^169^ Despite initial investigations into drugs based on the tau hypothesis not yielding promising results,^152^ numerous treatments are still actively being developed. These include kinase inhibitors, tau aggregation inhibitors, tau immunotherapies, antisense oligonucleotides that inhibit tau production, agents that promote autophagy-mediated degradation, and tau-targeted PROTACs.^166,170^

### Neuroinflammation hypothesis

Neuroinflammation is generally characterized as a chronic inflammatory response in the central nervous system (CNS) that fails to resolve on its own. It often involves the activation of glial cells and the release of pro-inflammatory factors during neuroinflammation.^171^ Microglia, the CNS foremost innate immune cells, acts as an initial defense against danger-associated molecular patterns and pathogen-associated molecular pattern receptors. Microglia are elongated, branched cells that monitor their environment and secrete neurotrophic factors in a state of homeostasis. Once stimulation is detected, microglia undergo morphological changes and initiate a variety of responses.^172,173^ Aβ is a typical trigger for microglial activation. Activated microglia migrate towards senile plaques, engulf Aβ, and release enzymes to break down Aβ. Over prolonged periods, they might become less efficient at handling Aβ but continue to generate proinflammatory cytokines.^174,175^ Aβ also causes the formation and activation of the NLRP3 inflammasome within microglia, which releases ASC specks that bind rapidly to Aβ in promoting Aβ aggregates and the spread of Aβ pathology.^176^ Interactions between microglia and tau protein in the later stages of AD may contribute to increased tau phosphorylation and exosomal tau secretion, thereby promoting the spread of tau.^177,178^ With the exaggerated activation, the complement cascade potentially leads to aberrant synapse pruning by microglia, further exacerbating AD pathology.^171^ Researchers have identified different activation stages of microglia, each associated with distinct gene expression patterns. Initial stages were characterized by genes related to cell proliferation, whereas later stages feature genes linked to immune responses.^171^ GWAS have pinpointed numerous risk genes closely linked to microglial activities, highlighting the significance of microglia as a promising therapeutic target.^179^ Targeting triggering receptor expressed on myeloid cells 2 (TREM2) has the potential to harness neuroprotective properties by elevating microglial responsiveness to pathological proteins.^180^ Meanwhile, APOE4 could modify the behavior and function of activated microglia, contributing to increased Aβ deposition, tau-associated neurodegeneration, enhanced inflammation, altered immune responses, and disrupted synaptic homeostasis.^123,181–184^ Consequently, diminishing APOE4 expression in Aβ plaque-associated microglia may offer an effective approach. In summary, neuroinflammation is intricately associated with Aβ and tau pathologies, and the discovery of numerous immune response-related risk factors indicates that neuroinflammation is a significant factor in AD pathogenesis. Recent investigations have also expanded the scope of AD-related inflammation, exploring how the gut microbiota, oral microbiome, and viruses such as herpesviruses and severe acute respiratory syndrome coronavirus 2 (SARS-CoV-2) impact neuroinflammation.^185–187^ Regarding anti-inflammatory therapies, the effectiveness of nonsteroidal anti-inflammatory drugs (NSAIDs) remains inconclusive.^188,189^ Despite this, the primary focuses in the development of anti-inflammatory drugs are appropriate intervention timing and enhancing target specificity.^171,190^ Currently, numerous drugs targeting inflammation-related receptors, signaling pathways, and pro-inflammatory cytokines are under clinical trials.^185^

### Oxidative stress hypothesis

During regular metabolic processes, the body produces reactive oxygen species (ROS), reactive nitrogen species, and other highly reactive and unstable substances. These substances are generally kept at low levels by an efficient antioxidant defense system to protect cells from oxidative damage.^191,192^ However, in the brain of AD patients, factors such as metal accumulation, overexpression of related enzymes (e.g., NADPH oxidase), and mitochondrial dysfunction are involved in producing excessive ROS, surpassing the ability of the endogenous antioxidant system and resulting in an oxidative imbalance. It will damage neuronal membrane lipids, proteins, and nucleic acids, ultimately causing neuronal cell death.^191,193–195^ The abnormality of the electron transport chain within mitochondria is particularly a significant contributor to free radical production. Aβ plays a crucial role in mitochondrial dysfunction by reducing the activities of key enzymes and disrupting the dynamics of mitochondria.^192,196^ Oxidative stress presented in the early stages of AD acts as a crosstalk between different hypotheses of AD.^197^ For example, oxidative stress modulates the process of APP and the activity of secretases, thereby promoting the amyloid pathway. Furthermore, it is instrumental in the phosphorylation of tau proteins and the subsequent formation of NFTs. The activation of microglia induced by ROS triggers a neuroinflammatory cycle. The presence of free metals and complexes of Aβ with metals act as catalysts for ROS production, ultimately leading to neuronal cell death.^195^ Given these connections between oxidative stress and other AD mechanisms, antioxidants have emerged as promising agents in AD treatment with positive outcomes observed in animal models.^198^ However, the efficacy of antioxidants in clinical trials for AD remains uncertain. Several studies have indicated that standalone treatments or treatments in combination with cholinesterase inhibitors did not confer significant cognitive benefits to patients with AD. Future efforts should focus on optimizing drug dosages and initiating antioxidant therapy early in the course of the disease’s progression for potentially improved outcomes.^199^ In summary, oxidative stress has garnered widespread attention as a significant factor in the pathogenesis of AD. Nevertheless, the interplay between Aβ and oxidative stress,^200^ as well as their sequence within AD,^201,202^ require further research and exploration.

### Metal ion hypothesis

In physiological conditions, trace metals maintain homeostasis of the neuronal metal ion microenvironment. This balance can be disrupted by the inappropriate deposition or misdistribution of metal ions, with the dyshomeostasis of Fe^2+^, Cu^2+^, and Zn^2+^ closely associated with AD.^203^ The accumulation of these biometals in Aβ plaques and NFTs plays a critical role in pathological protein deposition. For instance, they may modulate the activity of essential enzymes, alter the conformation of proteins, or disrupt clearing pathways.^203–205^ When metals are sequestered in protein deposits, it may initiate a cascade of ROS production and accentuate toxicity.^206^ Specifically, iron-induced oxidative stress causes increased release of iron from iron-containing proteins, converting Fe^3+^ to Fe^2+^ intracellularly. Fe^2+^ overload can induce ferroptosis and lipid peroxidation through the generation of ROS via the Fenton reaction, ultimately resulting in neuronal death. Similarly, Cu^+^ directly binds to lipoylated dihydrolipoyl transacetylase (DLAT), inducing lipoylated DLAT aggregation and ultimately leading to cuproptosis.^203^ The sequestration in protein deposits also causes functional metal loss, potentially contributing to the cognitive decline in AD. Zinc could interfere with signaling through N-methyl-D-aspartate (NMDA) receptors. Supplementation of zinc may promote the maturation of proBNDF, reducing synaptic dysfunction and neuronal death.^204,205^ Hence, zinc deficiency is crucial in the context of glutamate excitotoxicity and synaptic dysfunction in AD. Overall, metal dyshomeostasis is closely linked to various events in AD such as amyloidosis, tauopathy, oxidative stress, and neuronal death. This hypothesis provides an alternative approach to understanding the pathogenesis of AD and detecting pathological changes. Further research is necessary to elucidate its role in AD. Additionally, metal ion chelators, developed based on this hypothesis, need to overcome challenges such as adverse events and poor blood-brain barrier (BBB) permeability to demonstrate their potential therapeutic value.^203^

### Glutamatergic excitotoxicity

Glutamate is the main excitatory neurotransmitter of glutamatergic neurotransmission in the CNS.^206^ Their receptors comprise ionotropic glutamate receptors, including NMDA receptors, α-amino-3-hydroxy-5-methyl-4-isoxazole propionic acid (AMPA) receptors, and kainate receptors, as well as metabotropic glutamate (mGlu) receptors.^207^ Glutamate mainly interacts with NMDA receptors to control the influx of sodium and calcium to neurons. Magnesium ions act to shut the NMDA receptor’s cationic channel and block the entry of ions into neurons under physiological conditions. However, in AD, there is an overstimulation of NMDA receptors, which results in the dislodgement of magnesium and permits an excessive entry of sodium and calcium ions.^208,209^ The entry of sodium into neurons causes their temporary swelling, while an increase in calcium levels initiates various Ca^2+^-dependent processes. These processes include the creation of ROS, disruption of mitochondrial function, and the activation of necrotic/apoptotic pathways, ultimately resulting in permanent excitotoxic damage to the neurons.^210,211^ Overall, pharmaceutical validation of the glutamatergic excitotoxicity hypothesis demonstrates the effectiveness of neurotransmitter regulation in improving cognitive symptoms. However, the limitations of neurotransmitter-based medications and the focus on other hypotheses appear to hinder further investigation into the mechanisms of excitotoxicity. The observed changes in the inhibitory neurotransmitter system, exemplified by γ-aminobutyric acid,^212^ and the potential for excitotoxicity to alter cognitive levels earlier than Aβ and tau pathologies,^209^ suggest that excitotoxicity might hold greater potential in AD treatment.

### Microbiota-gut-brain axis hypothesis

In recent years, the microbiota-gut-brain axis hypothesis has attracted significant attention, unveiling potential pathways for novel therapeutic strategies.^213^ The microbiota predominantly consists of bacteria, with smaller populations of fungi, viruses, archaea, and protozoa. These microorganisms offer trophic and protective effects in metabolism and innate immunity and influence brain function via the gut-microbiota-brain axis.^214–216^ The microbiota-gut-brain axis refers to a bidirectional communication system between the gut and the brain, including metabolic, endocrine, neural, and immune pathways that can work independently or in concert.^213,216^ Alterations in the host’s diet, use of antibiotics, exposure to psychosocial stress, or irregularities in the immune system may shift the relative proportions of bacterial species, resulting in a disruption of the microbiota’s composition and functionality as dysbiosis.^214^ Subsequently, the intestinal epithelial barrier is compromised. Harmful substances and microorganisms in the intestinal tract could enter the bloodstream, triggering an immune response that may lead to systemic inflammation. The onset of systemic inflammation may allow inflammatory mediators to cross over the BBB and impact microglia, further exacerbating neuroinflammation.^213,217^ This process is accompanied by imbalanced neurotransmission,^218^ which ultimately leads to neuronal degeneration and damage. Overall, the microbiota-gut-brain axis hypothesis establishes a connection between the peripheral immune system and the CNS, offering a fresh perspective for AD research. Moreover, drugs and biomarkers^219^ related to the gut microbiome are potentially considered. However, the investigation of this mechanism is still in an early stage. The exact mechanisms by which the gut microbiome affects brain activity or its connections with other pathological features of AD remain unclear.

### Abnormal autophagy

Autophagy, a highly conserved metabolic degradation process, maintains cellular homeostasis by delivering intracellular protein aggregates and damaged organelles to lysosomes for degradation and recycling.^220,221^ It primarily occurs via three types: microautophagy, chaperone-mediated autophagy, and macroautophagy (commonly referred to as autophagy).^222^ Microautophagy is the simplest pathway in which cytoplasmic substrates enter vesicles formed by morphological changes in lysosomal or endosomal membranes, and are ultimately degraded within the lysosome.^220,223,224^ Chaperone-mediated autophagy involves chaperone proteins recognizing and binding to specific protein sequences (KFERQ-like motifs), facilitating substrate transfer to lysosomes through interactions with lysosomal membrane proteins (LAMP2A).^224–226^ Macroautophagy, the main subtype, is primarily regulated by mTORC1 for activating the unc-51-like autophagy activating kinase 1 (ULK1) complex and dephosphorylating transcription factor EB (TFEB) to induce autophagy. Under the regulation of autophagy-related protein complexes, a phagophore forms and gradually expands to a sealed autophagosome. The autophagosomes then move retrogradely along microtubules to the microtubule organizing center, which is rich in lysosomes. They fuse with lysosomes to form autolysosomes, where substrate degradation occurs. In certain instances, autophagosomes could first merge with endosomes to form amphisomes, which then fuse with lysosomes.^222,224,227–229^ However, the abundant accumulation of autophagic vacuoles in swollen (malnourished) neurons is observed to have a linkage with Aβ/APP-βCTF, suggesting that autophagy clearance is severely disrupted under pathological conditions and is closely linked to amyloid pathology.^115,225,230^ This makes autophagy a focal point in recent AD pathogenesis research. There is increasing evidence indicating that genetic factors, reduced expression of related proteins, and defective vesicular transportation are potential causes of autophagy pathway disruptions. These disruptions interfere with clearance mechanisms involving substrate engulfment, autophagosome formation, autophagosome-lysosome fusion, and lysosomal structure and function.^227,229^ In AD, autophagy defects mediate the disruption of protein homeostasis networks (production and extracellular secretion of Aβ, abnormal aggregation of tau protein) and lead to the accumulation of damaged organelles, such as dysfunctional mitochondria.^231^ In summary, abnormalities of autophagy are intimately related to the onset and progression of AD. There is a growing emphasis on the involvement of chaperone-mediated autophagy,^232^ contributions of glial cell autophagy,^233,234^ and the precise causes of mitochondrial autophagy disorders.^235^ Autophagy-stimulating drugs including small molecule therapies and gene therapies, have shown significant neuroprotective potential in various AD animal models, suggesting a potential intervention option.^220,222,231,236,237^ However, the challenges posed by the broad targets of autophagy modulators, and lack of appropriate in vivo autophagic flux detection methods, hinder further clinical applications of these drugs.^222,227^

## Signaling pathways linked to AD pathogenesis

### Neuroinflammatory signaling

Several pathological factors in AD, such as Aβ, pro-inflammatory cytokines, and oxidative stress, activate microglia and initiate downstream signaling pathways such as MAPK, NF-κB, and PI3K/Akt. The activation of these pathways further promotes the activation of microglia and the production of inflammatory mediators, exacerbating neurotoxicity.^238–240^ ERK, JNK, and p38 MAPK are three primary MAPK signaling pathways that may activate transcription factors such as AP-1 and NF-κB to release pro-inflammatory cytokines like TNF-α, IL-1β, and NO.^241,242^ NF-κB can be co-regulated by multiple pathways including MAPK and PI3K/Akt to enhance transcriptional activity, thus promoting the expression of pro-inflammatory and pro-oxidant enzyme genes.^239,243,244^ A recently identified microRNA, miR-25802, found to be overexpressed in AD, likely plays a crucial role in exacerbating disease pathology. This microRNA may regulate the polarization of microglial cells towards a pro-inflammatory phenotype through the modulation of the KLF4/NF-κB signaling pathway. Such alterations can further aggravate key pathological features in the 5xFAD mouse model including increased deposition of Aβ plaques and deficits in learning and memory.^245^ The NF-κB signaling pathway significantly impacts the expression of components related to the NLRP3 inflammasome, such as NLRP3 protein, ASC, pro-IL-1β, and pro-IL-18. The NLRP3 inflammasome activates caspase-1 through its assembly and activation processes. Activated caspase-1 can cleave gasdermin D (GSDMD), triggering pyroptosis and releasing IL-1β, IL-18, and ASC specks into the extracellular environment. This may exacerbate the spread of inflammation and neuronal death.^246–249^ Additionally, the connection between NF-κB signaling and NLRP3 inflammasome activation with AD tau pathology has garnered significant attention. Inactivated NF-κB pathways in microglia may reduce the seeding and amplification of tau proteins in microglia, thus rescuing cognitive deficits in young PS19 mouse models, yet the accumulation of tau inclusions in neurons of aged PS19 mice warrants further investigation.^250^ According to recent studies, pro-inflammatory cytokines like IL-1β may induce an increase in tau transcription in human primary neurons by activating the NF-κB signaling pathway in neurons. Brain-derived tau proteins may activate the inflammatory response in microglia via the TLR2/MyD88/NF-κB pathway.^251^ Research by Ising et al. suggests that tau proteins can activate the NLRP3 inflammasome, which then promotes excessive tau phosphorylation and aggregation by affecting specific tau kinases and phosphatases.^252^ These findings reveal the complex interplay between inflammatory responses and tau pathology, providing a more comprehensive understanding of AD’s molecular mechanisms. The activation of the cGAS-STING signaling pathway in AD also plays a crucial role in neuroinflammation. Studies by Xie et al. found that the abnormal accumulation of double-stranded DNA in the cytoplasm may bind to the cytoplasmic DNA sensor (cGAS), thereby specifically triggering the STING-interferon (IFN) signaling pathway in microglia, promoting the expression and secretion of inflammatory cytokines. The relationships between microglia and other cells, such as astrocytes and neurons, further extend the scope of inflammation, forming a complex network of inflammatory regulation.^253,254^ It is noteworthy that persistent neuroinflammation may lead to the infiltration of peripheral immune cells (such as T cells, B cells, monocytes, and neutrophils), yet the mechanisms of this infiltration and impacts on AD’s disease progression remain to be studied.^254–256^ A recent study using a special 3D human neuroimmune axis model explored the interactions between infiltrative peripheral immune cells and innate immune cells in AD. The study found that C-X-C motif chemokine ligand 10 (CXCL10) and its receptor CXCR3 play key roles in regulating the infiltration of CD8+ T cells into the brain, and the infiltrated CD8+ T cells appear to interact with microglia to jointly promote AD’s neurodegeneration.^257^ In the APP-PS1 transgenic mouse model, Unger et al. found that CD8+ T cells might affect brain activity by regulating genes associated with neuronal and synaptic functions, providing new clues about the potential mechanisms of CD8+ T cells in AD neuronal dysfunction and cognitive deficits.^258^ Additionally, TREM2 has emerged as a potential therapeutic target due to its potential role in early AD in modulating neuroinflammation, Aβ plaque deposition, and cognitive abilities.^259^ Recent research findings continue to reveal the potential mechanisms by which TREM2 plays a neuroprotective role in AD. For instance, Wang et al. suggest that the anti-inflammatory mechanisms induced by TREM2 may be associated with the PI3K-Akt-FoxO3a axis. The PI3K/Akt pathway, upregulated by TREM2, may regulate the activity and subcellular localization of FoxO3a, thereby reducing the expression levels of pro-inflammatory cytokines.^259^ Moreover, TREM2 has been reported to bind with high affinity to C1q (the initiator of the classical complement pathway) to effectively inhibit the classical complement pathway, protecting synapses from abnormal phagocytosis and loss in AD.^260^

### Lysosomal dysfunction

Lysosomes rely on a rich array of acidic hydrolases to selectively degrade and recycle both intracellular and extracellular materials, playing a crucial role in maintaining cellular homeostasis.^261^ Lysosomal dysfunction is considered a critical factor in the development of many diseases,^261^ which may manifest as impaired acidification, abnormal expression of lysosomal enzymes, lysosomal membrane stability issues, transport defects, and defects in autophagosome/endosome-lysosome fusion. These issues may disrupt lysosomal degradation pathways, including the autophagy-lysosomal pathway and endosomal-lysosomal system, leading to the accumulation of pathological proteins and damaged organelles, further disrupting the cellular environment.^261–263^ A key factor affecting lysosomal function is the pH controlled by the vacuolar (H+)-ATPase (V-ATPase), which uses the energy from ATP hydrolysis to drive H^+^ from the cytoplasm into the lysosome. Other factors such as Cl^-^, Ca^2+^, and Na^+^ ion channels/transporters also interact with the luminal pH and collectively regulate the lysosomal acidic environment.^264,265^ In AD, lysosomal acidification deficits may weaken the clearance of Aβ, ultimately leading to the accumulation of extracellular Aβ plaques.^115^ This phenomenon indicates that lysosomal-related clearance system dysfunction might be one of the early events in the progression of AD and has become a focus of current AD research. It has been reported that the PS1 holoprotein may facilitate N-glycosylation of the V0a1 subunit of V-ATPase and its trafficking from the endoplasmic reticulum (ER) to lysosomes, thereby promoting the assembly and maturation of V-ATPase.^266^ However, there are inconsistent views on a series of events caused by defects in PS1, including impaired maturation of V0a1 in lysosomes, V-ATPase dysfunction, and lysosomal acidification defects.^267,268^ Calcium dysregulation associated with PS1 has been proposed as a potential cause of endolysosomal defects.^268^ Lee et al. once again affirmed the link between lysosomal acidification dysfunction and V-ATPase, further elucidating that aberrant lysosomal acidification mediates transient receptor potential cation channel mucolipin subfamily member 1 (TRPML1) overactivation, resulting in dysregulation of lysosomal calcium ions. Moreover, they demonstrated that solely reversing lysosomal calcium ion levels in cellular models failed to impact lysosomal acidity and autophagic function beneficially.^269^ Another study suggested that PS1 mutations may lead to the opening of another calcium ion channel, two pore segment channel 2 (TPCN2), whose markedly enhanced activity greatly promotes lysosomal calcium efflux and lysosomal alkalinization.^270^ Thus, the relationship among PS1 gene mutations or deficiencies, lysosomal acidification, and lysosomal calcium ion dysregulation warrants further investigation. Recent research has also revealed the impact of other AD-related genes on lysosomal dysfunction. For instance, increased phosphorylation of APP β-C-terminal fragment (βCTF) Tyr682 inhibited the assembly and activity of V-ATPase by binding to the V0a1 subunit, resulting in elevated lysosomal pH and impaired degradation capacity.^271^

### Cholesterol metabolism

Cholesterol is abundant in the brain, serving as a critical component of the myelin sheath and the membranes of neural cells, including neurons and glial cells.^272^ The balance between cholesterol synthesis, transport, metabolism, and clearance is crucial for neuronal growth, synaptic plasticity, and learning and memory functions.^273–275^ In AD, cholesterol biosynthesis and catabolism are impaired, contributing to the progression of AD through mediation of Aβ, tau, inflammation, and other pathological changes.^275,276^ The connection between cholesterol and Aβ may be related to lipid rafts, which are cholesterol-rich microdomains on the plasma membrane. These rafts may facilitate the colocalization of APP with its cleaving enzymes, enhance the activities of β and γ secretases, and influence the endocytosis of APP, thereby mediating its amyloidogenic pathway.^276,277^ With the assistance of cholesterol transporter APOE, astrocyte-derived cholesterol could be transferred to neuronal membranes, regulating cholesterol-dependent lipid clusters (also known as lipid rafts) on neurons to promote Aβ generation. Differences in cholesterol levels caused by different APOE isoforms may be related to their cellular expression and regulatory mechanisms.^278^ Additionally, different APOE isoforms have varying impacts on Aβ pathology. Compared to APOE3 and APOE2, APOE4-mediated pathways of Aβ clearance are impaired, and APOE4 exhibits a higher affinity interaction with Aβ, potentially driving a more severe Aβ plaque burden,^119,121,123^ making it one of the strongest genetic risk factors for AD. Cholinergic dysregulation associated with ApoE4 also contributes to tau pathology. For instance, in chimeric human cerebral organoids (chCOs), astrocytes and neurons carrying the APOE4 genotype could jointly promote tau phosphorylation in neurons, closely linked to the role of APOE4 in increasing cholesterol levels and lipid droplet content, suggesting that APOE4 may affect tau phosphorylation in AD by influencing lipid metabolism.^279^ Litvinchuk et al. revealed a potential synergistic effect between APOE4 and tau pathology, wherein APOE4 may induce the abnormal accumulation of certain cholesterol esters in glial cells. This accumulation subsequently triggers the activation of glial cells, the release of inflammatory cytokines, infiltration of T-cells, and synaptic damage.^280^ Furthermore, activation of the inflammation-related NLRP3 inflammasome signaling pathway in different types of neural cells was closely associated with high cholesterol load, which triggered neuroprotective properties in activated microglia but promoted oxidative stress in neurons, further enhancing the expression of NLRP3 inflammasomes, inducing neuronal pyroptosis, and impairing the phagocytic capacity of microglia.^281^

### Mitochondrial dysfunction

Mitochondria are the primary source of cellular energy and mediate a multitude of biological processes including biosynthesis, redox balance, calcium signaling, and apoptosis, serving as the core drivers of vital activities.^282,283^ Observations in AD-afflicted brains of regionally reduced glucose metabolism and alterations in several mitochondrial enzyme activities suggest mitochondrial dysfunction.^284^ This is primarily manifested by defects in energy metabolism, increased oxidative stress, calcium ion imbalance, and abnormal mitochondrial dynamics, all potentially leading to neuronal dysfunction and even apoptosis, exacerbating the neurodegenerative changes in AD.^282,285^ Moreover, AD pathological biomarkers could directly impact mitochondrial function, creating a vicious cycle. Aβ inhibits the activity of key mitochondrial enzymes such as electron transport chain enzyme complex IV, pyruvate dehydrogenase (PDH), and α-ketoglutarate dehydrogenase (αKGDH), reducing the efficiency of electron transfer, diminishing ATP synthesis, and stimulating the production of ROS.^286^ Additionally, Aβ interacts specifically with mitochondrial Aβ-binding alcohol dehydrogenase (ABAD), impeding the binding of NAD to ABAD and inducing ROS production.^287,288^ The generation of ROS and the imbalance of the antioxidant system further damage mitochondrial DNA, lipids, and proteins, aggravating mitochondrial dysfunction and cellular apoptosis.^283,289^ As the most common secondary messenger in cells, the importance of calcium ions is self-evident, and their homeostatic disruption is a significant factor in mitochondrial damage.^290^ Aβ may increase cytosolic calcium levels and impair mitochondrial calcium buffering functions through various pathways including plasma membrane receptors and calcium channels,^291^ enhanced ER calcium release,^292^ and the mitochondrial inner membrane calcium channel MCU.^293,294^ This leads to mitochondrial calcium overload, causing cyclophilin D (CypD) to relocate from the mitochondrial matrix to the inner membrane, promoting the formation of the mitochondrial permeability transition pore (mPTP), further inhibiting ATP synthesis, activating oxidative stress, and apoptosis.^289,295^ Moreover, tau is also associated with mitochondrial calcium imbalance, and due to the critical role of tau in microtubule structure and function, its abnormal phosphorylation and aggregation may adversely affect mitochondrial axonal transport, impacting local metabolic needs and overall neuronal function.^296,297^ Impairments in mitochondrial fission and fusion mechanisms, as well as mitophagy, are also areas of concern in AD. Alterations in the expression levels of proteins related to fission/fusion processes (such as Opa1, Drp1, MFN1/2, Fis1)^298^ and post-translational modifications of Drp1^299,300^ may bias mitochondria towards excessive fission, increasing mitochondrial fragmentation, leading to damage in mitochondrial energy biology and accumulation of mitochondrial DNA damage.^283,301^ Fragmented mitochondria significantly obstruct mitophagy in AD, where PINK1/parkin-regulated mitophagy is a focal point of current research.^302–304^ PINK1 accumulates on the outer membrane of damaged mitochondria and activates parkin, which then ubiquitinates several mitochondrial outer membrane proteins to initiate the autophagic pathway, engulfing damaged mitochondria to maintain mitochondrial health and function.^305^ PINK1/parkin cascades related to Aβ, APP-CTFs, tau, and the APOE4 isoform could lead to the accumulation of damaged mitochondria.^306^ The accumulation of Aβ and increased p-tau, synaptic dysfunction, in turn, negatively regulate mitophagic activity, accelerating the pathological progression of AD.^304^

### Calcium signaling

Intracellular calcium could originate from the opening of plasma membrane calcium channels, such as voltage-gated and ligand-gated calcium channels, and the release of organelles like the ER and mitochondria.^307–309^ Calcium plays a multifaceted role in regulating gene expression, neurotransmitter release, membrane excitability, and inducing synaptic plasticity.^310,311^ Additionally, plasma membrane calcium ATPases (PMCA), the sarco/ER calcium ATPase (SERCA), the sodium-calcium exchangers (NCX), and Ca^2+^-binding proteins also regulate cytosolic calcium concentration.^312–315^ Maintaining this calcium homeostasis is fundamental to calcium signaling, and disruption in cytosolic calcium concentration gradients, as well as abnormalities in calcium signaling pathways, may lead to neurodegenerative diseases such as AD and Parkinson’s disease (PD), cardiovascular diseases, and metabolic disorders.^315–318^ In AD, enhanced activity of L-type VGCCs, potentially related to their interaction with Aβ/tau, promotes excessive calcium influx into cells.^319^ Studies have shown that using L-type calcium channel blockers could mitigate the upregulation of L-type VGCCs and abnormal calcium influx induced by Aβ.^320^ Ligand-gated calcium channels such as NMDAR and α7nAChR, highly permeable to Ca^2+^, are closely associated with Aβ.^308^ Overactivation of NMDARs by Aβ leads to abnormal calcium influx, triggering a cascade of downstream signaling events, resulting in dendritic spine loss, reduced distribution of NMDARs on neuronal membranes, impaired synaptic plasticity, and ultimately, cognitive decline.^321,322^ Complexes formed by Aβ with α7-nAChR efficiently promote Aβ internalization and increased calcium influx, further affecting extracellular Aβ plaque accumulation and synaptic transmission.^308^ Abnormal intracellular calcium signaling could also impact various organelles such as the ER, mitochondria, and lysosomes. The impaired function of SERCA and/or overactivation of calcium release channels (InsP3R and ryanodine (RyR) receptors) on the ER could facilitate the activation of the ER stress response.^307^ The ER regulates the expression of unfolded protein response (UPR)-related target genes by increasing the formation of transcription factors ATF4, XBP1, and ATP6, providing cellular stress tolerance. However, persistently high-stress levels may trigger ER-mediated apoptosis.^323^ Mitochondrial physiological functions are closely linked to calcium transfer between the ER and mitochondria, a process crucially mediated by MAMs.^324–326^ Under the influence of Aβ, the expression of some MAM-related proteins, such as IP3Rs and VDAC1, is significantly increased,^325,327,328^ leading to mitochondrial Ca^2+^ overload, inhibition of normal ATP synthesis, and potential release of apoptotic signals.^329^ Research has found that lysosomal acidity is also within the realm of calcium regulation, where excessive Ca^2+^ released from the ER-resident RyR receptor can impair the function of lysosomal V-ATPase, causing lysosomal acidification defects, reducing lysosomal protease activity, and leading to the accumulation of p-tau.^330^

### Insulin signaling

Insulin regulates glucose metabolism, neuronal growth and survival, synaptic plasticity, and cognition,^331–333^ functions closely linked to two main insulin signaling pathways: phosphatidylinositol 3-kinase (PI3K)-Akt and Ras/Raf-MAPK.^334,335^ The PI3K-Akt pathway is a crucial component of insulin signaling, and in AD brains, there is observed a decrease in IRS-associated PI3K activity and reduced phosphorylation of Akt kinase.^336,337^ Lower levels of Akt activation weaken the inhibition of glycogen synthase kinase-3 (GSK-3), which in turn positively affects the phosphorylation of tau protein and the production of Aβ.^333,338,339^ mTORC1, a downstream molecule of Akt, also serves as a critical nexus linking insulin signaling with the autophagy system. Its role in the inhibitory phosphorylation of IRS1, synaptic protein synthesis, synaptic plasticity, and autophagy regulation is significantly correlated with the accumulation of pathological protein aggregates and impaired learning and memory functions in AD. Some drugs targeting mTORC1 have been demonstrated in animal studies to effectively inhibit abnormal mTORC1 activation, thereby enhancing autophagy, reducing Aβ and tau pathology, and helping to delay cognitive decline. However, some studies express divergent views on the activity of mTORC1 in AD.^340^ Furthermore, the increased production of inflammatory mediators like TNF-α and the activation of stress kinases such as JNK, PKR, and IKK could promote the inhibitory serine phosphorylation of IRS-1, downregulate insulin signaling in the brain, and induce AD neurological dysfunction.^331,341^

### Dysregulated neurotrophic signaling pathway

Neurotrophic factors not only promote the survival, growth, and differentiation of neurons but are also crucial for maintaining synaptic plasticity and neuronal signaling functions.^342,343^ In AD, key neurotrophic factors include NGF and brain-derived neurotrophic factor (BDNF), which exert their effects through specific receptors such as tropomyosin-related kinase (Trk) and p75^NTR^.^15^ In AD, there is a reduction in the conversion of proNGF to mature NGF and an enhancement in the degradation of mature NGF,^344^ leading to a deficiency in mature NGF and accumulation of proNGF in the brain. The lack of mature NGF may promote the phosphorylation of APP at T668, reducing its binding to TrkA and affecting its subcellular localization, thus increasing amyloidogenic processing of APP and Aβ production.^345^ The accumulation of proNGF and downregulation of TrkA (pro-survival signal) levels favor the predominance of pro-apoptotic signaling mediated by p75^NTR^, further promoting the degeneration of basal forebrain cholinergic neurons.^346,347^ Downregulation of BDNF expression leads to weakened BDNF signaling in AD.^348^ This weakened signaling triggers the activation of JAK2/STAT3 and C/EBPβ signaling pathways in the AD brain and inhibits downstream Akt signaling molecules,^349^ thereby promoting the activation of asparagine endopeptidase (AEP; also called δ-secretase) to cleave APP and tau proteins.^350,351^ The cleaved tau fragments could bind to TrkB receptors, further inducing neuronal apoptosis.^349^ A study suggested that impaired BDNF nutritional signaling also stimulated the expression of APP and PS1 to exacerbate amyloidogenesis.^352^ Similarly, Aβ can interfere with common neuroprotective signaling pathways, such as the Raf-MAPK/ERK pathway and the PI3K-Akt pathway, initiated by the binding of BDNF to TRKB, inducing cortical neurons into a dysfunctional state.^353^ According to recent research, microglial repopulation/self-renewal contributed to the restoration of BDNF expression and activation of the BDNF/TrkB neurotrophic signaling pathway, significantly reversing cognitive deficits in 5xFAD mice. This suggests that BDNF may provide potential benefits for AD treatment through its positive modulation of impaired synaptic plasticity and cognitive memory.^354^

### BBB dysfunction

**Fig. 4:**
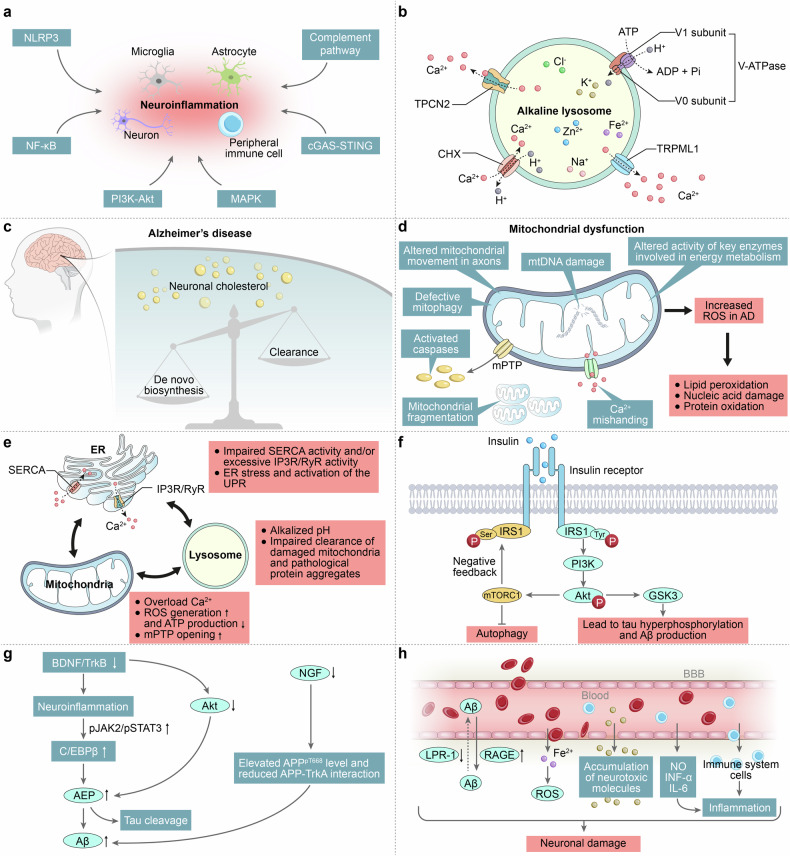
Signaling pathways linked to AD pathogenesis. **a** Neuroinflammatory signaling. It involves interactions among various cell types, which influence neuroinflammation by activating multiple pathways. This leads to the production of inflammatory mediators and neuronal damage, accelerating the pathological progression of AD. **b** Lysosomal dysfunction. It may be related to impairments in V-ATPase-mediated lysosomal acidification and/or dysregulation of lysosomal calcium homeostasis. However, the specific mechanisms require further investigation to be definitively determined. **c** Aberrant cholesterol metabolism. **d** Mitochondrial dysfunction. Mitochondria in AD are damaged in various ways, including impairments in oxidative phosphorylation, calcium homeostasis, mtDNA, mitochondrial fusion and fission, axonal transport, and mitophagy. These dysfunctions lead to impaired energy production and increased oxidative stress.^283^ **e** Calcium signaling in AD. Under physiological conditions, calcium ions follow a strict concentration gradient. In AD, the elevated cytosolic calcium concentration and calcium-responsive signaling cascades adversely affect protein folding in the ER, energy production in mitochondria, and lysosomal acidity.^307^ **g** Insulin signaling in AD. **f** Dysregulated neurotrophic signaling pathway. **h** BBB dysfunction. The disruption of the integrity and alterations in the transport functions of BBB lead to the abnormal entry and exit of certain substances into and out of brain tissue, resulting in neuronal damage and further exacerbating the pathological progression of AD^644^

The BBB is formed by components such as endothelial cells, astrocytes, and pericytes, along with the basement membrane, and together with other cells like microglia and neurons, they constitute the neurovascular unit (NVU).^355,356^ The BBB not only allows highly selective permeability of substances entering and exiting through specialized structures (seal off adjacent BECs) but also dynamically regulates cerebral blood flow through the process of neurovascular coupling, maintaining homeostasis and neuronal function in the CNS.^355,357–359^ Dysfunction of the BBB includes disruption of BBB integrity (or BBB leakage), changes in BBB transport functions, reduced cerebral blood flow, and neuroinflammation. Some evidence suggests that in AD, dysregulation of tight junction proteins, increased matrix metalloproteinase signaling, and degeneration and loss of pericytes may all contribute to BBB leakage, leading to the accumulation of numerous blood-derived neurotoxic proteins in the brain, causing neuroinflammation and oxidative stress.^356,360–362^ Disruption of the BBB may also lead to ischemic/hypoxic brain damage and increase Aβ production.^358^ Abnormal expression of transport proteins/receptors in the BBB, such as downregulation of LRP1 which exports Aβ from the brain to the blood, impaired function of Pgp, and upregulation of RAGE that facilitates the entry of Aβ from the blood into the brain, could be potential reasons for impaired Aβ clearance and substantial accumulation in the brain.^363^ Reduced activity and expression of the GLUT-1 transporter in the BBB suggest decreased glucose uptake and utilization by the brain,^360,363^ which may further exacerbate cerebrovascular degeneration, BBB breakdown, and Aβ pathology in models overexpressing APP, inducing neurodegeneration and cognitive deficits (Fig. 4).^364^

## Clinical trials of AD

### Biomarkers for AD diagnosis

The National Institute on Aging and Alzheimer’s Association (NIA-AA) proposed a research framework to define the biology of AD using Aβ deposition, pathologic tau, and neurodegeneration AT(N) biomarkers.^365^ The current established biomarkers mainly include imaging biomarkers, cerebrospinal fluid (CSF) biomarkers, and blood biomarkers. Molecular imaging techniques like magnetic resonance imaging (MRI) and positron emission tomography (PET) are commonly used to detect structural and functional brain activity in vivo.^366^ Specifically, structural MRI (sMRI) assesses hippocampal and entorhinal cortex atrophy in the medial temporal lobe, ^18^fluorodeoxyglucose (^18^FDG)-PET detects reduced glucose metabolism in the posterior cingulate and temporoparietal lobes, and PET imaging shows Aβ and tau deposition.^366–368^ However, sMRI and (^18^FDG)-PET indicate neurodegeneration or neuronal injury in the AT(N) framework with limitations in specifically diagnosing AD. They cannot accurately differentiate AD from other neurodegenerative diseases with similar pathologies, such as frontotemporal degeneration and TDP-43 proteinopathies with medial temporal lobe atrophy. Additionally, the atypical AD and cerebrovascular diseases may also complicate the diagnosis.^2,369–371^ Therefore, these methods typically need to be combined with other clinical information and assessment tools for a comprehensive evaluation of AD pathology. Amyloid PET and tau PET not only reflect the overall accumulation and spatial distribution of amyloid plaques and NFTs but may also detect abnormal brain changes earlier than neurodegeneration, thus providing opportunities for early intervention in the disease.^366,371^ Studies have reported that amyloid PET exhibits 90% sensitivity and specificity in diagnosing AD, and tau PET can specifically identify AD dementia from other neurodegenerative diseases, showing higher diagnostic accuracy than MRI markers.^368^

NIA-AA’s AT(N) research framework includes CSF biomarkers such as Aβ_42_ (or the Aβ_42_/ Aβ_40_ ratio), phosphorylated tau (P-tau), and total tau (T-tau). Notably, P-tau181 concentration is the most accurate indicator for differentiating AD from non-AD dementia.^372,373^ While amyloid and tau PET and CSF biomarkers specifically indicate AD-related pathology, they are not entirely equivalent. Studies show a highly negative correlation between amyloid PET and CSF results, whereas CSF P-tau and tau PET findings are inconsistent. This discrepancy is related to their respective representations of PHFs formation and pathological tau deposition, with the latter’s higher correlation to cognitive abilities supporting tau PET as the most effective method for predicting cognitive decline in AD.^365,374^ A recent study indicated that within 20 years, abnormalities in CSF Aβ_42_, the ratio of CSF Aβ_42_ to Aβ_40_, CSF P-tau181, CSF T-tau, CSF neurofilament light chain (NfL), and hippocampal volume (as detected by sMRI) appear in sequence before the clinical diagnosis of SAD.^375^ This suggests that CSF biomarkers may reveal changes in the disease process earlier than imaging biomarkers.^7^ Therefore, selecting effective and reliable biomarkers, considering their sensitivity and specificity, as well as the potential inconsistencies among different biomarkers, is crucial for determining the nature and pathological stage of the disease in clinical practice. Recently, more CSF biomarkers reflecting other biological processes in AD have emerged, such as axonal injury and synaptic dysfunction (NfL, neurogranin (NG), synaptosomal-associated protein 25, visinin-like protein 1),^366,367,372^ neuroinflammation (TREM2, YKL40, S100B, glial fibrillary acidic protein (GFAP)),^371,376–378^ changes in neurotrophic protein levels (BDNF and NGF),^379^ BBB disruption (soluble platelet-derived growth factor receptor-β),^380^ and metabolic changes (sphingomyelin, ceramide, fatty acid-binding protein 3, ubiquitin C-terminal hydrolase L1).^381,382^ Extracellular vesicles (EV), crucial in AD pathology spread, have gained attention. Proteomic studies found elevated C1q levels in MCI and AD groups, and increased CatB concentration in CSF Aβ_42_-positive cases. These factors are potentially involved in early AD pathology through synaptic aberrant pruning and rapid abnormal metabolism of APP, respectively. They present potential CSF EV-related biomarkers pending further validation.^383,384^ Blood biomarkers offer an economical, convenient, minimally invasive, and highly accessible diagnostic alternative.^385–387^ Many CSF biomarkers (like Aβ, P-tau, NfL, GFAP) also show promising applications in blood, with advancements in highly sensitive analytical platforms and detection techniques enhancing diagnostic precision and reliability.^368,388,389^ For instance, an innovative integrated proteomic assay accurately measured levels of 21 AD-related blood biomarkers, which jointly evaluated AD from five dimensions: neurodegeneration, inflammation, innate immunity, vascular function, and metabolic activity. Machine learning models built on this dataset have accurately classified AD/MCI and Aβ pathology across different ethnicities, demonstrating potential benefits in early disease screening, pathology progression monitoring, and assessing the clinical efficacy of treatments.^390^ In summary, the emergence of AT(N) and non-AT(N) biomarkers has significantly improved the accuracy of AD diagnosis. The use of “composite biomarker panel”^390^ (effective combination of biomarkers) could comprehensively reflect the biological state of AD and enhance diagnostic accuracy. This is of great importance for differentiating MCI/AD patients from cognitively normal individuals, distinguishing AD from other neurodegenerative diseases, and even identifying AD subtypes. However, AD-related comorbidities may reduce the diagnostic value of biomarkers.^391–393^ For example, coexisting αSyn pathology in AD correlates with lower CSF P-tau181 and NG levels,^394^ while comorbidity like hypertension lowers plasma Aβ concentration but increases plasma P-tau181 and P-tau217 levels.^388,395^ Future research should focus on developing more AD-specific biomarkers while also identifying biomarkers for non-AD-related diseases, aiding in a clearer understanding of AD pathology and accurately distinguishing AD from other neurodegenerative diseases.^368^

### Clinical drugs

**Fig. 5:**
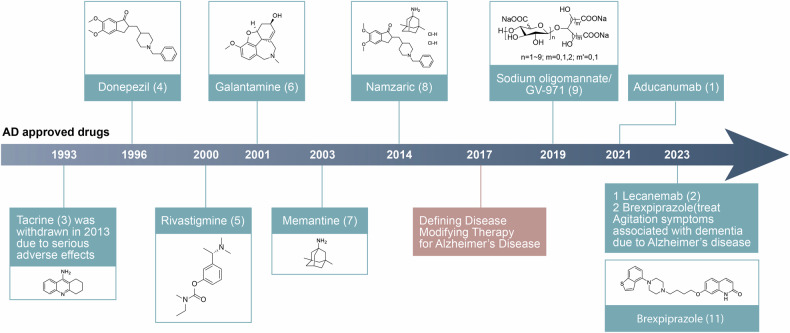
Approved drugs for AD by FDA/China. Notably, the definition of disease-modifying therapies, capable of producing enduring and impactful changes in the clinical progression of AD, was first proposed in 2017.^412^ (The numbers **1,** **2**,…… **8,** **9** in the figure represent the drug identifiers defined by the authors)

Traditional AD drugs (Fig. 5) are categorized into two classes: AChEIs (tacrine (**3**), donepezil (**4**), rivastigmine (**5**), galantamine (**6**)) and NMDA receptor antagonists (memantine (**7**)).^396^ AChEIs boost postsynaptic stimulation by increasing both the level and the action duration of ACh, thereby enhancing cognitive and behavioral functions in patients.^397^ Tacrine (**3**) was approved for AD treatment in 1993 and pulled from the market in 2013 due to its liver toxicity. Nevertheless, it has potential in the study of multitarget-directed ligands.^30,398,399^ Second-generation AChEIs, including donepezil (**4**), rivastigmine (**5**), galantamine (**6**), are more selective. They exhibited fewer side effects or improved pharmacokinetic profiles, establishing them as first-line drugs for AD.^98,400^ Although these drugs have been widely used, ongoing research focuses on optimizing dose, dosage form, routes of administration, and combination therapies to minimize adverse effects and improve patient compliance as much as possible.^401–403^ The donepezil (**4**) transdermal patch, named Adlarity, was FDA-approved in 2022 for treating mild, moderate, and severe dementia of the Alzheimer type.^404^ Its weekly dosing frequency showed bioequivalence to daily oral administration at the same dosage while presenting fewer gastrointestinal adverse events than oral administration. This also offers greater convenience compared to the once-daily rivastigmine (**5**) patch.^405^ The application of nanocarriers is also being explored to deliver these cholinesterase inhibitors through intranasal administration, intravenous injection, and other methods. Nanocarriers play a crucial role in increasing drug concentrations, slowing drug release, and achieving excellent bioavailability.^401,406,407^ Furthermore, the combination use of appropriate cholinesterase inhibitors, such as donepezil (**4**) and galantamine (**6**), or the combination of cholinesterase inhibitors with other neurologic drugs, metal chelators, or antioxidants, may yield surprising effects in the management of cholinergic drugs in AD, including efficacy, tolerability, and safety.^402,408^ Memantine (**7**) is an FDA-approved NMDA receptor antagonist for the treatment of moderate to severe stages of AD. It modulates glutamate transmission and dopamine receptors, exhibiting certain efficacy in improving patients’ cognitive function, daily living abilities, and behavior.^409,410^ Namzaric (**8**, fixed-dose combination memantine (**7**) extended-release/donepezil (**4**)) also provides another treatment option for patients with moderate to severe AD.^51^ These drugs primarily function by modulating neurotransmitter levels but cannot alter the course of the disease,^409,411^ which are instructive for designing new drugs. In 2017, a review^412^ proposed “disease modifying therapy for AD”, which aims to intervene in the fundamental biological mechanisms to halt the disease’s progression and provide enduring therapeutic benefits to patients. Sodium oligomannate (**9**, GV-971), an oligosaccharide extracted from marine algae, was conditionally approved in China in 2019 amidst ongoing debates regarding its mechanism of action and therapeutic efficacy.^54,413^ Sodium oligomannate (**9**, GV-971) was postulated to counteract AD by inhibiting neuroinflammation triggered by gut dysbiosis and disrupting the formation of Aβ fibrils.^56,414^ Further research indicated that sodium oligomannate (**9**, GV-971) altered the composition and abundance of the gut microbiome in a sex-dependent manner in both APPPS1-21 and 5xFAD models. This modulation influenced microbial metabolism and peripheral inflammation, regulated the activation state and functionality of microglia, and thereby reduced neuroinflammation and amyloidosis.^415^ Currently, two phase IV clinical trials (NCT05181475 and NCT05058040) are ongoing to further investigate its efficacy and safety, with an expected continuation until 2025. Aducanumab (**1**), lecanemab (**2**), and donanemab (**10**) are monoclonal antibodies targeting Aβ, each of which has met with differing outcomes: Aducanumab (**1**)^416,417^ received controversial FDA accelerated approval in 2021; Lecanemab (**2**)^61^ gained traditional approval in 2023; Donanemab (**10**)^63^ has completed phase III trials and is in the process of market authorization. Their status is closely linked to their mechanisms. Aducanumab (**1**) binds to 3-7 amino acids of Aβ, targeting soluble oligomers and insoluble fibrils.^418,419^ Lecanemab (**2**), associated with the E22G Aβ,^420^ showed stronger binding to soluble Aβ aggregates (oligomers and protofibrils) than aducanumab (**1**).^421^ Donanemab (**10**) targets pyroglutamate-modified Aβ, binding specifically to plaques.^419^ All three have shown efficacy in clearing Aβ plaque and slowing cognitive decline, but the risks of amyloid-related imaging abnormalities (ARIA) and their treatment costs are noteworthy.^422–424^ Brexpiprazole (**11**), commonly prescribed for depression and schizophrenia, targets serotonin, dopamine, and norepinephrine receptors. It is known to help mitigate agitation in individuals with AD.^425–427^ These innovative medicines delve deeper into AD mechanisms and present diverse target choices, holding the potential to halt or reverse AD progression. Further studies are needed to understand drug mechanisms, assess long-term efficacy, and ensure safety. In addition, the unfavorable risk-benefit ratio in AD makes drug repurposing a common approach. The long, high-cost, and resource-heavy process of developing AD medications, coupled with their high rate of failure, has led to growing interest in repurposing medications originally designed for other conditions, including cancer, cardiovascular diseases, psychiatric disorders, diabetes, and other neurological diseases.^428,429^ These drugs are noted for their extensive safety and tolerance profiles, as well as their potential for multiple uses.^428,430^ Additionally, the advancement of artificial intelligence (AI)-based computational tools is facilitating drug repurposing, presenting a promising strategy AD drug development.^431–433^

As documented on ClinicalTrials.gov, the AD research landscape encompasses 187 clinical trials, spanning phase I, II, and III, specifically targeting AD and MCI attributed to AD. Among these trials, 36 drugs are in phase III, 87 in phase II, and 31 in phase I.^434^ The major mechanisms of action center around: 1) neurotransmitter receptors, including AChE, NMDA receptor, 5-hydroxytryptamine receptor, nicotinic α7 receptor, and adrenoceptor; 2) Aβ, including the reduction of Aβ production (such as γ-secretase inhibitors and modulators, BACE1 inhibitors, and α-secretase activators), prevention of Aβ aggregation, and enhancing Aβ clearance (vaccines and antibodies); 3) tau proteins (phosphorylation modulators, aggregation inhibitors, microtubule stabilizers, antibodies, and vaccines); and 4) inflammation (NSAIDs, microglia modulators).^434–437^ The majority of phase II and III trials center around neurotransmitter receptors and Aβ mechanisms, while tau and inflammation drugs are more prominent in phase II, often featuring repurposed compounds. Typical/Representative AD drugs in advanced clinical stages are detailed in Table 1. Semagacestat (**12**, LY-450139) was the first γ-secretase inhibitor to enter phase III clinical trials. A clinical trial (NCT00594568) aimed at assessing the long-term progression of AD found deterioration in cognitive and functional status across all trial groups. Additionally, participants experienced adverse reactions such as gastrointestinal symptoms, skin cancer, and infections, which are speculated to be related to the inhibition of other γ-secretase substrates, including notch, CD44, ErbB4, and cadherin.^438–441^ Avagacestat (**13**, BMS-708163) is an orally administered γ-secretase inhibitor that exhibited greater selectivity for APP-C99 compared to semagacestat (**12**, LY450139).^440^ Phase I studies indicated its effectiveness in reducing Aβ levels. However, during a phase II study assessing its safety and tolerability in patients with prodromal AD (NCT00890890), adverse events including gastrointestinal issues and skin cancer were observed in the high-dose treatment group.^442^ Researchers have explored inhibiting β-secretase (BACE1) as an alternative to γ-secretase inhibitors due to its higher selectivity for APP, aiming to reduce Aβ production.^443^ Umibecestat (**14**, CNP520), a fourth-generation BACE1 inhibitor, initially showed good safety and tolerability in early clinical studies.^444,445^ However, two phase II/III trials (NCT02565511 and NCT03131453), conducted on older individuals with high risk of AD (carriers of the APOE4 allele) but without cognitive impairment, were terminated prematurely. This decision was made due to observations of mild cognitive decline and brain atrophy in participants.^446,447^ Elenbecestat (**15**, E2609), a fourth-generation BACE1 inhibitors, was among the last BACE1 inhibitors to reach phase III clinical trials.^448^ A phase III trial (NCT02956486) assessing effectiveness and safety in early-stage AD patients was terminated due to an unfavorable risk-benefit ratio. More specifically, literature^446,449^ indicates that the termination was due to the lack of help in cognition and the emergence of side effects such as nightmares, weight loss, rash, and liver damage. ALZ-801 (**16**), an orally administered small molecule drug with tramiprosate as its active ingredient, exhibited effective anti-Aβ oligomer activity without binding to plaques, potentially reducing the risk of ARIA associated with plaque clearance.^450,451^ In interim results from its phase II trial (NCT04693520), the drug lowered biomarker levels and showed the potential to slow the decline in memory and learning abilities in early AD patients carrying the APOE4 gene (either APOE4/4 or APOE3/4).^425^ The ongoing phase III clinical trial (NCT04770220) aims to further validate these positive results regarding efficacy and safety in APOE4 homozygous individuals with early AD, with the study expected to continue until 2024. Varoglutamstat (**17**, formerly PQ912), the first small molecule glutaminyl cyclase inhibitor to enter phase II clinical trials, targets an enzyme that catalyzes the conversion of glutamate to pyroglutamate at the N-terminus of Aβ. This modification results in Aβ forms that are more prone to form toxic aggregates.^452,453^ In its phase IIa study (NCT03919162), varoglutamstat (**17**, formerly PQ912) demonstrated acceptable safety and tolerability, as well as a reduction in working memory decline.^454^ The ongoing phase IIb VIVIAD trial (NCT04498650) aims to further explore its long-term safety, tolerability, effects on cognition, and impact on AD biomarkers.^455^ Solanezumab (**18**, LY2062430) is an antibody targeting the intermediate domain of Aβ, effective against soluble, monomeric, non-fibrillar forms of Aβ, thus promoting the dissolution of plaques.^456^ In the initial two phase III trials (NCT00905372 and NCT00904683) evaluating the drug’s efficacy compared to a placebo in patients with mild to moderate AD, the drug did not significantly delay cognitive or functional decline. However, it appeared to potentially alter the disease course in patients with mild AD. In the expedition3 trial (NCT01900665), aimed at further validating the drug’s efficacy in patients with mild AD, the drug was declared unsuccessful in 2016, as it failed to meet its primary endpoints.^457–459^ Gantenerumab (**19**) is a subcutaneously administered antibody capable of binding to two regions of Aβ – the N-terminal and the central structural domain.^460^ It targets soluble oligomers, protofibrils, and plaques.^461^ Two phase III trials (NCT03444870 and NCT03443973) were recently terminated. In these trials, when assessing the efficacy and safety of gantenerumab (**19**) in participants with early (prodromal to mild) AD, the drug showed little clinical benefit in slowing cognitive decline, potentially due to limited clearance of amyloid plaques, with 5.0% participants experienced amyloid-related imaging abnormalities-effusion (ARIA-E) related side effects.^461,462^ Tideglusib (**20**), a non-ATP-competitive GSK-3β inhibitor, exhibits neuroprotective and anti-inflammatory properties.^463^ In its phase II study (NCT01350362), which evaluated the drug’s efficacy, safety, and tolerability in patients with mild to moderate AD, it did not meet some primary and secondary endpoints.^464^ TRx0237 (**21**, LMTX) is a tau aggregation inhibitor.^465^ All phase III trials have now been completed or terminated. Two earlier studies (NCT01689233 and NCT01689246) conducted on participants with mild AD and mild to moderate AD, respectively, indicated that the drug demonstrated good safety and potential benefits as a monotherapy.^466,467^ Another phase III trial (NCT03446001) aimed to further confirm the safety and efficacy of 16 mg/day monotherapy compared to placebo in participants with mild to moderate AD, with results pending disclosure.^468^ Bepranemab (**22**, UCB0107), an antibody targeting the central region of tau, potentially inhibits tau aggregation and propagation.^469^ A phase II study (NCT04867616) for AD is undergoing to evaluate its efficacy, safety, and tolerability in patients with MCI or mild AD. E2814 (**23**) is a monoclonal antibody that targets the tau microtubule-binding region, thereby inhibiting tau protein aggregation and seed propagation.^470^ The drug is currently undergoing three clinical trials. A phase I/II trial (NCT04971733) aims to assess the safety, tolerability, and target engagement of E2814 (**23**) in participants with dominantly inherited AD (DIAD), with completion expected in 2025. The other two phase II/III trials (NCT01760005 and NCT05269394) aim to evaluate the efficacy of the combination of E2814 (**23**) and lecanemab (**2**) in early-onset AD. These trials respectively use the changes in cognitive measures and tau PET as their primary outcome measures and are expected to conclude in 2027. AADvac1 (**24**) is the first tau vaccine to enter clinical trials,^469^ aiming to inhibit tau aggregation, remove tau aggregates, prevent pathological spread, and slow disease progression. A phase II study (NCT02579252) evaluating the drug’s safety and efficacy in patients with mild AD showed that AADvac1 (**24**) was well-tolerated with no significant adverse reactions. However, its clinical efficacy requires further validation.^471^ NE3107 (**25**, formerly HE3286) is a small insulin sensitizer that inhibits inflammation.^425^ A phase III clinical trial (NCT04669028) has been completed, aimed at testing the safety and efficacy of the drug in elderly patients with mild to moderate AD. The results indicated that the drug was well-tolerated and effectively slowed down the rate of cognitive decline in participants, significantly improving cognitive function.^472^ ALZT-OP1 (**26**) is a combination treatment of cromolyn sodium and ibuprofen. It induces the transformation of microglial cells into a pro-phagocytic/neuroprotective activation state and blocks Aβ aggregation.^473^ ALZT-OP1 (**26**) has completed a phase III study (NCT02547818) assessing its safety and efficacy in subjects with evidence of early AD. The study aimed to determine whether the combination therapy of ALZT-OP1 (**26**) could slow down or reverse cognitive and functional decline in early-stage AD participants. AL002 (**27**) is a TREM2-specific monoclonal antibody that activates TREM2 to enhance microglial function, thereby reducing Aβ plaque formation and attenuating neurite dystrophy.^474^ A phase II study (NCT04592874) is currently underway to evaluate the efficacy and safety of AL002 (**27**) in participants with early-stage AD. Masitinib (**28**) is a potent and selective tyrosine kinase inhibitor targeting multiple aspects of AD, including inhibition of microglia and mast cell activation, modulation of Aβ and tau protein signaling pathways, and prevention of synaptic damage.^475^ It is currently undergoing a phase III clinical trial (NCT05564169). The objective of this study is to confirm the efficacy of masitinib (**28**) as an adjunct therapy to cholinesterase inhibitors and/or memantine (**7**) in improving cognitive and functional abilities in patients with mild to moderate AD.^476^ Repurposed drugs include nilvadipine (**29**), a calcium channel blocker for the treatment of hypertension, and pioglitazone (**30**), a drug initially developed for diabetes. Nilvadipine (**29**) displays various properties, such as decreasing Aβ production, increasing cerebral blood flow, and exerting anti-tau and anti-inflammatory activities. A phase III trial (NCT02017340) testing the efficacy and safety of nilvadipine (**29**) in participants with mild to moderate AD indicated that, while the drug demonstrated good safety, it did not show significant benefits in slowing cognitive decline in AD patients.^477^ Pioglitazone (**30**) is a PPARγ agonist widely used in the treatment of T2D.^478^ Two phase III clinical trials (NCT01931566 and NCT02284906) assessed the safety and efficacy of the drug in participants with AD-induced MCI but were terminated due to insufficient efficacy.

**Table 1:** Representative AD drugs in late clinical stages against different target types (sourced from https://clinicaltrials.gov) (The numbers **12,** **13**,…… **30** in the table represent the drug identifiers defined by the authors)

Clinical drug	Chemical structure	Target type	Sponsor	NUT identifier	Phase	Status
**Semagacestat (12, LY-450139)**		γ-secretase inhibitor	Eli Lilly and Company	NCT00594568	III	Completed
**Avagacestat (13, BMS-708163)**		γ-secretase inhibitor	Bristol-Myers Squibb	NCT00890890	II	Terminated
**Umibecestat (14, CNP520)**		BACE1 reversible inhibition	Novartis Pharmaceuticals	NCT02565511 NCT03131453	II/III II/III	Terminated Terminated
**Elenbecestat (15, E2609)**		BACE1 reversible inhibition	Eisai Co., Ltd.	NCT02956486	III	Terminated
**ALZ-801 (16)**		Prevent Aβ_42_ from forming oligomers	Alzheon Inc.	NCT04770220	III	Active, not recruiting
**Varoglutamstat (17)**		Glutaminyl cyclase inhibitor	Vivoryon Therapeutics N.V.	NCT03919162 NCT04498650	II II	Active, not recruiting Completed
**Tideglusib (20)**		Tau protein kinase inhibitor with neuroprotective and anti-inflammatory effects	Noscira SA	NCT01350362	II	Completed
**TRx0237 (21)**		Tau aggregation inhibitor	TauRx Therapeutics Ltd.	NCT01689233 NCT01689246 NCT03446001	III III III	Completed Completed Completed
**NE3107 (25, formerly HE3286)**		Reduces inflammation	BioVie Inc.	NCT04669028	III	Completed
**ALZT-OP1 (26)**		Promote microglia recruitment to plaques, and phagocytosis of Aβ deposits	AZTherapies, Inc.	NCT02547818	III	Completed
**Masitinib (28)**		Targets activated cells of the neuroimmune system (mast cells and microglia)	AB Science	NCT05564169	III	Not yet recruiting
**Nilvadipine (29)**		Calcium channel blocker	Prof Brian Lawlor	NCT02017340	III	Completed
**Pioglitazone (30)**		PPARγ agonist	Takeda	NCT01931566 NCT02284906	III III	Terminated Terminated
**Solanezumab (18, LY2062430)**		Anti-amyloid monoclonal antibody	Eli Lilly and Company	NCT00905372 NCT00904683 NCT01900665	III III III	Completed Completed Terminated
**Gantenerumab (19)**		Anti-amyloid monoclonal antibody	Hoffmann-La Roche	NCT03444870 NCT03443973	III III	Terminated Terminated
**Bepranemab (22, UCB0107)**		Anti-tau monoclonal antibody	UCB Biopharma SRL	NCT04867616	II	Active, not recruiting
**E2814 (23)**		Anti-tau monoclonal antibody	Washington University School of Medicine	NCT01760005 NCT05269394	II/III II/III	Recruiting Active, not recruiting
**AADvac1 (24)**		Anti-tau vaccine	Axon Neuroscience SE	NCT02579252	II	Completed
**AL002 (27)**		Anti-TREM2 monoclonal antibody	Alector Inc.	NCT04592874	II	Active, not recruiting

In summary, the development of AD drugs has faced numerous challenges. Factors contributing to the suboptimal performance of drugs include the selection of drug targets, the use of biomarkers and animal models in experimental designs, and other issues such as late treatment initiation, dose-dependent side effects, challenges in BBB permeability, and the heterogeneous presentation of patients.^182,479,480^ In the extensively researched Aβ hypothesis, Aβ stands as the most direct drug target. However, the structural polymorphism of Aβ, including monomers, soluble oligomers, protofibrils, and amyloid plaques, along with numerous pathogenic variants, complicates the selection of precise targets and adds to the complexity of designing effective drugs.^481^ When Aβ antibodies, such as bapineuzumab (**31**), did not yield significant therapeutic effects, research shifted towards inhibiting the formation of Aβ.^109,170^ However, the side effects associated with targeting β- and γ-secretases arise because these enzymes have a wide range of substrates that are vital in other physiological processes.^170^ In addition, the overemphasis on the Aβ hypothesis has also hindered the emergence of diverse new targets.^482,483^ Biomarkers play a crucial role in patient selection, biological effect detection, dose optimization, and monitoring response progress, with recent approvals of Aβ monoclonal antibodies benefiting from new and accurate biomarkers.^83,423^ The disparity in drug performance between preclinical and human trials has driven the evolution of animal models. Current AD animal models have shifted from single genetic mutation models to multi-gene transgenic models, and consider non-genetic pathogenic factors and species differences to more accurately simulate the AD progression process.^484–487^ While immunotherapy appears to be the most advanced therapeutic strategy, primarily targeting traditional targets such as Aβ and tau, a noticeable paradigm shift is occurring toward small-molecule therapeutic modalities.^435^ These modalities, characterized by their simplicity, maturity, and adaptability, provide a promising avenue for emerging targets. The development of a new generation of small-molecule drugs for AD is thus an exciting prospect. Furthermore, diverse mechanisms of inhibition, including selective, dual-targeted, allosteric, covalent, PROTACs, and PPI-targeted approaches, are enhancing drug-like properties, safety, and efficacy. This multifaceted approach aims to expedite the development of valuable drugs for both traditional and emerging targets, streamlining the drug development cycle and mitigating associated challenges.

## Potential therapeutic drugs

The multifactorial nature of AD onset, coupled with the complex interactions among these factors, poses significant challenges to drug development. The limited efficacy of traditional medications, combined with the high failure rates in clinical drug development due to insufficient efficacy or adverse effects, has raised the bar for the development of the next generation of AD drugs. These drugs aim to furnish a repertoire of diverse and precise treatments tailored to individual patients and their distinct pathological processes. Progress in understanding the pathophysiological mechanisms, combined with advancements in drug development technologies, has paved the way for the discovery of novel drugs. Details of next-generation compounds in AD are outlined in Table 2.

**Table 2:** Development of next-generation compounds in AD (The numbers **32,** **33,** **34**…… in the table represent the compound identifiers defined by the authors)

Drug	Target	In vitro/in vivo model	Results	Reference
kadsuranin [(+)-2] (**32**) gomisin N [(−)-2] (**33**)	GSK-3β	ICR mice, Aβ-induced SH-SY5Y cell injury model, APP/PS1 double transgenic mice	Increased the liver glycogen level, inhibited the p-GSK-3β/p-tau signaling pathway, alleviated the cognitive disorders in AD mice	492
**34**	GSK-3β	SH-SY5Y neuroblastomas	Decreased the levels of hyperphosphorylated tau	495
**35**	GSK-3α	P10 rats	Lowered the tau phosphorylation at pThr231	497
OCM-51 (**36**)	GSK-3β	Pluripotent stem cells with induced expression of Ngn2 transgene	Decreased the levels of phosphorylated tau	499
dp-FINDY (**37**)	Dyrk1A	HEK293 cells	Decreased Dyrk1A	503
**38**	HDAC6	Aβ_25−35_-induced mice	Protected against memory dysfunction	513
**39**	HDAC6	SH-SY5Y and Neuro-2a cells treated with Aβ_1−40_ or transfected with pCAX APP 695 and pRK5-EGFP-Tau P301L plasmids, scopolamine-treated rats, 3xTg-AD mice	Inhibited tau phosphorylation and aggregation, ameliorated impaired learning and memory	514
**43** **44**	JNK3	3xTg mice, APPswe/PS1dE9 mice	Slowed down the decline in cognitive memory	519
**45**	AChE and BuChE	APPswe/PS1dE9 mice	Rescued learning and memory impairments	530
F681-0222 (**47**)	AChE and BACE1	APPswe/PS1dE9 mice	Decreased soluble Aβ_42_ levels	534
**48**	AChE and GSK-3β	Mouse neuroblastoma N2a-Tau cells, scopolamine-treated ICR mice	Inhibited tau hyperphosphorylation, ameliorated the cognitive disorders	536
**49**	AChE and GSK-3β	Neuroblastoma N2a cells, neuroglia BV2 cells, scopolamine-induced mice	Decreased tau phosphorylation level, reduced oxidative stress, slowed down the cognitive decline	537
**50**	AChE and PDE4D	BV-2 cells, Aβ_25-35_ induced PC12 nerve cells	Inhibited inflammation	540
**52**	α7 nAChR	Time-delay and scopolamine-induced amnesia mice	Reversed the deficits of short-term episodic and working memory	552
**55**	M1 mAChR	Mouse cortical neurons	Showed less agonism	554
PS48 (**57**)	PDK-1	SH-SY5Y cells, rat primary cortical neuron exposed to the longchain saturated fatty acid	Activated Akt, reversed the inhibition of LTP	556
ACD856 (**58**)	Trk	Scopolamine-induced AD mice, C57BL/6 J mice	Attenuated the memory-impairing effects, restored cognitive function	557
**59**	GSK-3β	Aβ_25-35_-induced SH-SY5Y cells, AlCl_3_ combined with _D_-galactose induced mice	Reduced the release of cytokines, reduced the expression of APP and p-tau, increased p-GSK-3β expression, exhibited behavioral performance superior to that of the model group	464
PT-65 (**62**)	GSK3	SH-SY5Y cells, HEK-293 T cells with overexpression of GSK3β, okadaic acid-induced mice	Degraded GSK3, attenuated GSK3-mediated tau hyperphosphorylation, ameliorated learning and memory impairments	576
ALI6 (**65**)	Aβ-LilrB2	Primary neurons treated with Aβ	Inhibited the binding of Aβ to neurons, restored p-cofilin/cofilin level and protected neurons	586
**66**	Aβ-LilrB2	SH-SY5Y cells	Reversed the cofilin dephosphorylation, tau hyperphosphorylation and neurites outgrowth inhibition induced by Aβ	587
iododiflunisal (**67**) luteolin (**68**) sulindac (**69**) olsalazine (**70**) flufenamic (**71**)	TTR-Aβ	SH-SY5Y cells	Reduced caspase-3 levels	589
NXPZ-2 (**72**)	Keap1-Nrf2	Aβ_1-42_-induced AD mice	Ameliorated learning and memory dysfunction	590
POZL (**73**)	Keap1-Nrf2	Primary cultured cortical neurons, transgenic APP/PS1 AD mouse	Decreased oxidative stress, slowed down the pathological progression of spatial learning and memory dysfunction	591

### Selective inhibitors

**Fig. 6:**
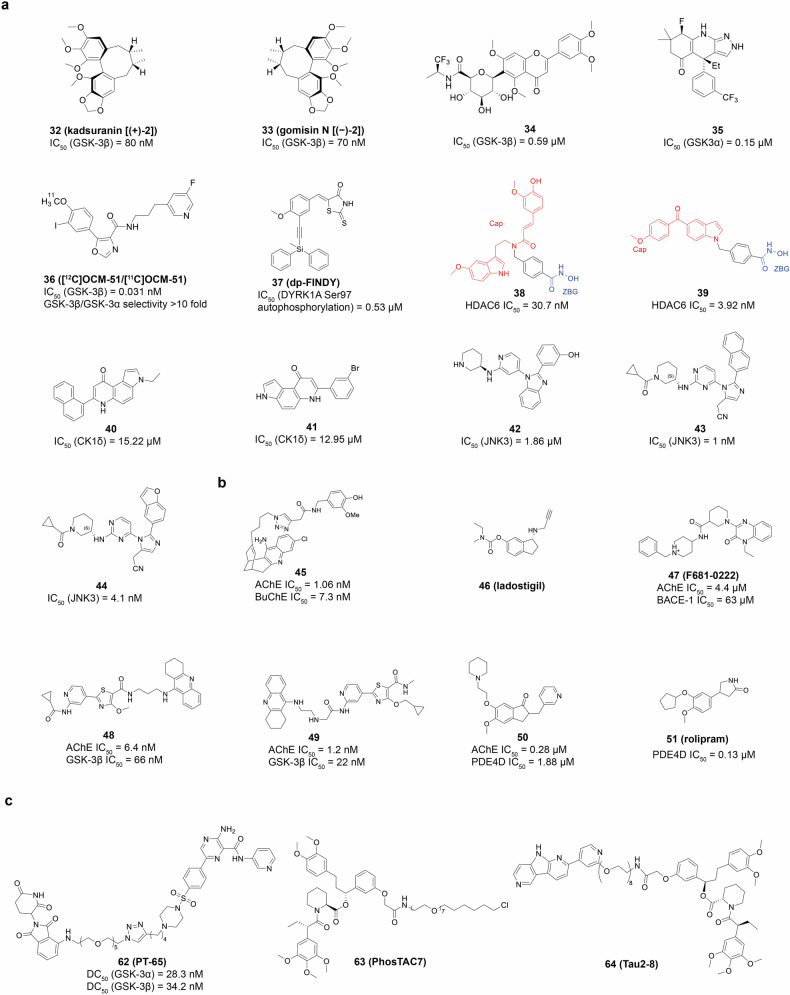
**a** Chemical structures of selective inhibitors **32**-**44**. **b** Dual-target inhibitors **45**-**50**. **c** GSK-3 degrader **62**, as well as PhosTACs **63** and **64**. (The numbers **32,** **33**,…… **51,** **62,** **63,** **64** in the figure represent the compound identifiers defined by the authors)

Given the association of pan-inhibitors with cytotoxicity and adverse events, coupled with a deepening understanding of the physiological functions of pathological proteins, the development of selective inhibitors has advanced significantly.^488–490^ These inhibitors are capable of specifically targeting categories, subtypes, and structural domains,^491^ potentially providing more pronounced benefits in terms of efficacy, safety, and tolerability.^67^ Kadsuranin [(+)-2] (**32**) and gomisin N [( − )-2] (**33**), which are two stereoisomers of schisandrin B extracted from the fruits of *S. chinensis*, have been shown to effectively inhibit GSK-3β in an ATP-competitive manner. Administering these compounds has been shown to effectively mitigate memory deficits and markedly reduce the expression of phosphorylated tau in the hippocampus in the APP/PS1 double-transgenic mice.^492^ Targeting less conserved substrate binding sites, as opposed to ATP binding sites, might offer advantages in terms of drug specificity, functional regulation, and safety.^493,494^ For example, compound **34** demonstrated these benefits.^495^ As the role of GSK-3α in promoting Aβ production and tau phosphorylation in AD models is recognized, selective inhibition of GSK-3α has emerged as a promising therapeutic strategy.^494,496,497^ The GSK-3α ATP-competitive inhibitor **35** could cross the BBB and significantly reduce tau phosphorylation at pThr231 in neonatal rat brains, potentially delaying early pathological progression in AD.^497^ It is noteworthy that simultaneous inhibition of both GSK-3α and GSK-3β could excessively activate the wnt/β-catenin pathway, leading to abnormal cell proliferation and other detrimental effects.^496,498^ Therefore, the ideal state for selective drugs is to ensure efficacy while providing a suitable therapeutic window for safety. For instance, the selective GSK3β inhibitor OCM-51 (**36**) could achieve a beneficial balance between reducing tau phosphorylation and preventing overactivation of the β-catenin signaling pathway at appropriate doses.^499^ Additionally, leveraging the dynamic changes of targets may be a potential strategy for developing selective inhibitors. Given that overexpression of dual-specificity tyrosine phosphorylation-regulated kinase 1 A (DYRK1A) may influence the initial progression of AD through mechanisms including the hyperphosphorylation of pathologically relevant substrates such as tau, APP, PS1, regulation of axonal transport of APP, and participation in the selective splicing of tau pre-mRNA,^500–502^ the compound dp-FINDY (**37**) effectively targets the spatial dynamic changes in the ATP-binding site between the DYRK1A folding intermediate and the folded state, specifically acting on the folding intermediate.^503^ This may reduce excessive interference with numerous physiological substrates of this target and offer a novel perspective in selective drug design. Histone deacetylases (HDACs) are epigenetic regulators that modulate gene expression by removing acetyl groups from lysine residues on proteins, affecting processes like cell proliferation, differentiation, and development.^504,505^ Among them, HDAC6 has two catalytic domains and a C-terminal zinc finger domain, interacts with tau and α-tubulin, and is involved in the degradation of protein aggregates, mitochondrial transport, and cognitive memory,^506–509^ making it relevant to AD pathology. HDAC6 inhibitors typically consist of three parts: a zinc-binding group (ZBG), a cap group, and a hydrocarbon motif connecting the cap and ZBG.^510,511^ Their selectivity often involves strong hydrophobic interactions between the cap group and a large surface area on HDAC6, known as the “L1 loop pocket”.^507,512^ Compound **38**, incorporating cap group of melatonin and ferulic acid, enhanced HDAC6 selectivity while providing significant antioxidant capacity, alleviating spatial working and non-spatial long-term memory deficits in Aβ_25-35_-injected mice at lower doses.^513^ Compound **39** achieved strong HDAC6 selectivity through interaction with another specific pocket on HDAC6, inhibiting tau hyperphosphorylation and aggregation. It demonstrated neuroprotective activity through ubiquitination mechanisms and improved learning and memory in animal models, presenting a potential therapeutic avenue for AD.^514^ In most cases of selective inhibitor development, research initially relies on the scaffold of lead compounds to provide basic affinity and molecular framework. Subsequent modifications enhance drug-target binding, solubility, metabolic stability, and BBB permeability. Compounds **40** and **41** were identified through a combination of docking-based virtual screening and pharmacophore modeling from an in-house oncology compound library. Their shared scaffold may offer new insights for casein kinase 1δ (CK1δ) inhibitor development.^515^ In AD, c-Jun N-terminal kinase3 (JNK3) activation is closely associated with neuronal damage, amyloid deposition, and the formation of tau tangles.^516^ Hah et al. have conducted in-depth studies on this target, continuously refining and developing several generations of compounds based on the structure of pan-JNK inhibitor **42**, which was identified through an in-house kinase-focused library screening. These compounds yielded significant improvements in potency, selectivity, and pharmacokinetic properties while maintaining key interactions with JNK3.^517–519^ Recently studied compounds **43** and **44** exhibited excellent performance in three behavioral tests of homozygous APPswe/PS1dE9 double transgenic mouse models and 3xTg mouse dementia models (Fig. 6a).^519^

The development of selective inhibitors benefits the understanding of the roles played by different targets and their subtypes in AD, and it may also reduce the risk of side effects. Some adverse effects may originate from the off-target proteins. Differences in amino acids, explicit water molecules, spatial conformation and dynamics between the target and other proteins binding sites could serve as the basis for drug selectivity. However, in AD drug development, designing inhibitors with high selectivity poses significant challenges when faced with highly conserved or homologous binding pockets. The discovery of additional pockets on the target enzyme, target optimization (identifying substitutable targets), and the use of computational tools may offer new strategies. Nevertheless, the complexity and diversity of AD mechanisms suggest the difficulties of targeting specific targets and their limited impact on the disease progression. In addition to targeting specific enzymes, drugs aim to improve efficacy and reduce adverse reactions by focusing on specific distribution and functions in the pathological stage. For instance, PROTAC technology leverages E3 ligases, which may be selectively expressed in certain tissues, to drive the targeted degradation of specific targets,^520^ offering significant opportunities for AD treatment. Covalent drugs also exhibit impressive performance in selective targeting,^521^ potentially providing novel inhibitory approaches for kinases such as CK1, which have previously only been targeted with non-covalent ATP competitive inhibitors.^522^ Further drug development techniques will also be discussed below, aiming to enhance drug efficacy and safety within a broader scope of selectivity.

### Dual-target inhibitors

Given the multifactorial nature of AD^523^ and the suboptimal effects of single-target drugs,^524^ the search for effective dual- or multi-target inhibitors has emerged as a new research trend. These inhibitors act on one or more targets with additive or synergistic effects, aiming to increase efficacy, prolong therapeutic effects, minimize side effects, and lower drug doses.^68,69,525^ Compared with combined therapies, they further reduce the risk of drug-drug interactions and simplify administration, making treatment safer, more effective, and more convenient for patients.^524,525^ From a biochemical standpoint, growing evidence supports a link between cholinergic abnormalities and other pathophysiological features of AD, including abnormal Aβ and tau. Consequently, cholinesterase inhibitors have become a fundamental approach in AD treatment.^526^ Targeting both AChE and Butyrylcholinesterase (BuChE) not only alleviates cognitive impairment in AD patients by increasing ACh levels but also serves as a disease-modifying agent, delaying the formation of Aβ plaques.^527–529^ The dual inhibitor of AChE and BuChE, compound **45**, significantly enhanced the learning and memory abilities of aged AD mice. The significant alleviation in Aβ burden, anti-inflammatory and antioxidative effects, and enhanced synaptic transmission activity were also observed in the hippocampus.^530^ Given the elevated activity of monoamine oxidase-B (MAO-B) observed in AD, dual inhibition of AChE and MAO-B holds promise for synergistic effects on cholinergic system recovery and Aβ plaque formation, along with potential benefits in alleviating oxidative stress injury.^531^ Ladostigil (**46**), an AChE/MAO-B inhibitor developed through a pharmacophore fusion strategy,^532^ has completed a clinical phase II trial (NCT01429623). The trial aimed to evaluate the safety and efficacy of low-dose ladostigil (**46**) in patients with MCI. The results indicated that the drug was well-tolerated and safe, seemingly possessing the potential to delay the progression of AD.^533^ Compound F681-0222 (**47**) leveraged the functional interplay between BACE1 and AChE to decrease soluble Aβ_42_ levels in the brain tissue of APPswe/PS1dE9 transgenic mice.^534^ The simultaneous modulation of AChE and GSK-3β has the potential on improving cholinergic and tau protein signaling pathways.^523,535^ AChE/GSK-3β inhibitors **48**^536^ and **49,**^537^ developed through a pharmacophore linkage strategy, exhibited promising results by significantly inhibiting tau hyperphosphorylation and ameliorating cognitive disorders in scopolamine-treated ICR mice. Additionally, inhibiting AD-related phosphodiesterases (PDEs) could consequently enhance synaptic transmission and mitigating cognitive deficiencies.^538,539^ Compound **50** is a dual-inhibitor of AChE and PDE4D. It exhibited exceptional neuroprotection against cell death and more substantial anti-neuroinflammatory effects in the hippocampus of AD model mice induced by Aβ_25-35_ than the combined treatment of donepezil (**4**) and rolipram (**51**) (Fig. 6b).^540^

For diseases with complex etiologies, single-target drugs often struggle to interfere with the complete network regulation of the disease and tend to produce significant toxicity. The design and application of dual-targeted and multi-targeted inhibitors place a greater emphasis on the interrelations of pathological factors, enhancing the convenience of medication for patients. Multi-target drugs can act on multiple interconnected targets in AD. Although their activity on a single target may be lower compared to single-target drugs, the synergistic effects of multi-target modulation result in a total effect greater than the sum of the individual effects, leading to better efficacy and fewer adverse reactions. The primary strategies include pharmacophore-linked and pharmacophore-merged methods.^541^ Although these approaches facilitate drug design on a technical level, relying on a limited set of known SARs for pharmacophores may somewhat limit the structural diversity of the drugs and narrow the range of targets. Inspiration for drug design often draws from natural products and computer-aided screening. Additionally, the physicochemical properties, pharmacokinetic characteristics, and toxicity of the drugs are critical factors that must be carefully considered during the design processes.

### Allosteric modulators

**Fig. 7:**
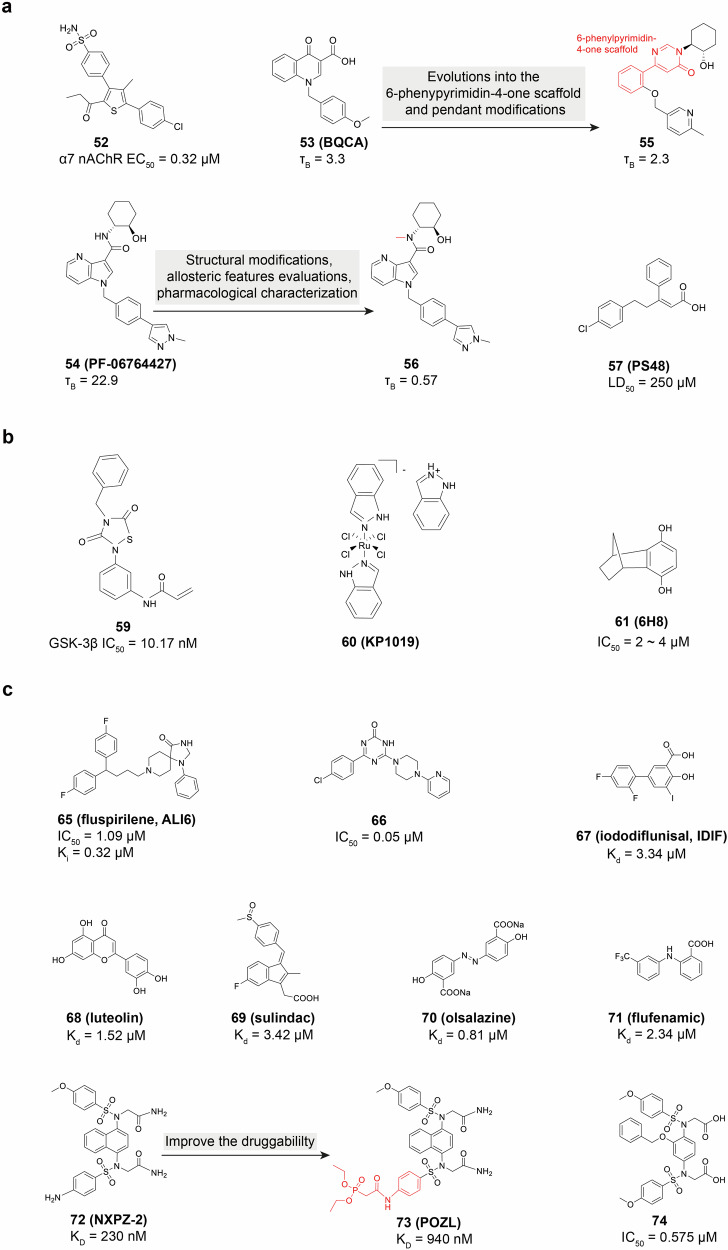
**a** Chemical structures and modification schemes of allosteric modulators **52-57**. **b** covalent inhibitors **59**-**61**. **c** Compounds **65**-**74** target the PPI network. (The numbers **52,** **53**,…… **57,** **59,** **60,** **61,** **65**,…… **74** in the figure represent the compound identifiers defined by the authors)

Allosteric modulators typically attach to regions distinct from the orthosteric site of receptors, inducing conformational changes to regulate the affinity and/or efficacy of orthosteric ligands, or to directly modulate receptor activity with positive, negative, or neutral effects.^542–545^ This precise tuning of receptor activity has revitalized the development of anti-γ-secretase drugs in the field of AD. Allosteric modulators of γ-secretase encourage the production of shorter, less toxic Aβ subtypes, and even potentially minimize effects on Notch and some other substrates. Some γ-secretase modulators (GSMs) also exhibited promising safety outcomes in preclinical studies and clinical trials.^546–548^ Compared to orthosteric sites, allosteric sites often have lower conservation and greater diversity,^549^ providing new avenues for drug development targeting highly homologous subtypes, such as nAChR and mAChR. The α7 nAChR subtype presents a potential approach for treating AD due to its high expression in cognitive function-related brain areas and interaction with Aβ.^550,551^ Selective positive allosteric modulators (PAMs) targeting the α7 nAChR subtype, such as compound **52**, slowed the decline of episodic/working memory in amnesia mouse models. Unlike orthosteric agonists, **52** did not cause receptor desensitization even with repeated dosing, and is currently being evaluated in clinical trials for its efficacy and safety in mild to moderate AD patients.^552^ M1-mAChR positive allosteric modulators (M1-PAMs), such as BQCA (**53**) and PF06764427 (**54**), achieve subtype selectivity through allosteric effects but have significant agonistic activity that may lead to side effects like diarrhea.^544,553^ The respective optimized derivatives of BQCA (**53**) and PF06764427 (**54**), compounds **55**.^554^ and **56,**^555^ require further in vitro and in vivo studies to evaluate their pharmacokinetic properties and allosteric modulation effects. Moreover, achieving signaling bias through allosteric modulation could enhance the safety of M1-mAChR drugs, making it a key consideration in the development of M1-mAChR allosteric ligands.^542,544,545^ Beyond the cholinergic system, allosteric drugs find broad application in AD. For example, chlorphenylalic acid PS48 (**57**) targets PDK-1 allosteric pocket to restore Akt insulin responsiveness. The drug reduced Aβ toxicity without over-regulating insulin signaling, presenting a promising strategy for AD prevention or treatment.^556^ In a phase I study (NCT05077501), the novel Trk receptor PAM ACD856 (**58**).^557^ demonstrated good safety and tolerability, as well as favorable pharmacokinetic properties, potentially benefiting neurotrophic factor signaling.^558^ Several reviews^70,559–561^ have extensively summarized allosteric modulation strategies targeting other proteins such as GSK-3β, NMDARs, AMPA receptors, and RIPK1 (Fig. 7a).

Allosteric modulation, with its distinctive features of low-conservation binding sites, subtype or even signaling pathway selectivity, saturated allosteric effects,^562^ and subtle-tuning of target function, exhibits strong appeal in AD drug development. Nonetheless, the discovery and development of allosteric drugs are facing challenges. Advantages of molecular docking and dynamics simulations, X-ray crystallography, and cryo-electron microscopy have facilitated the discovery of allosteric sites to enhance our understanding of allosteric modulation.^563,564^ However, the complexity of allosteric modulation requires a number of in vitro and in vivo studies to thoroughly assess and analyze the functional effects of compounds and the factors influencing their characteristics.^564^ Clearly, the potential benefits for AD cognitive deficits and the safety of allosteric drugs still need broader experimental data to support further optimization.^544,546^

### Covalent inhibitors

Covalent inhibitors, which form covalent bonds with their target proteins, rely on the specificity and stability of these interactions to exhibit superior potency, selectivity, and duration of action. This mechanism offers patients a convenient therapeutic option.^521,565^ Based on experiences in cancer treatment and other diseases, the development of AD covalent drugs also has a broad prospect. In cancer therapy, covalent inhibitors often target cysteine residues with acrylamide warheads.^565–567^ Based on this, compound **59**, which features an acrylamide warhead, can covalently bind to cysteine in GSK-3β. It significantly reduced the expression of APP and p-tau in the hippocampus of AD mice and improved spatial learning and memory abilities.^464^ A widely studied Ru(III) anticancer drug, KP1019 (**60**), reveals a unique anti-Aβ strategy. Unlike conventional methods that inhibit Aβ production and aggregation, KP1019 (**60**) counteracted Aβ toxicity to neuronal cell models by promoting the formation of soluble high-molecular-weight Aβ aggregates.^568^ This suggests that metal-based covalent inhibitors have promising potential in AD drug development. The electrophilic warheads and targeting residues of covalent inhibitors are continuously being developed. For example, the 6H8 (**61**) fragment, obtained through NMR screening from the Maybridge library, may act as a covalent warhead targeting the pathological substrate APP of γ-secretase, thereby hindering Aβ production.^569,570^ This could be a supplementary method to avoid potential side effects of γ-secretase inhibitors.^569^ In summary, the application of covalent inhibitors to some undruggable targets (such as Aβ, tau, and APPTM) has broadened the possibilities of drug design. The characteristics of covalent inhibitors are expected to reduce the required dosage and frequency of administration, thereby improving patient compliance and offering a new strategy for AD treatment. However, the potential toxicity of covalent inhibitors has always been a concern. Improving the selectivity of covalent inhibitors is critical and can be optimized through various means, including adjusting the reactivity and reversibility of the electrophile (warhead),^571,572^ non-covalent scaffolds, dosage, etc. Relevant literature has discussed these aspects (Fig. 7b).^565,567,573^

### PROTACs

The ubiquitin-proteasome system (UPS) is one of the primary protein degradation pathways within the cell. However, in AD, the dysfunction of this clearance pathway becomes a significant contributor to the accumulation of pathological proteins.^574^ The PROTACs exploit the UPS system to precisely target specific proteins, improving the accuracy and speed of protein degradation.^575^ Various reviews^574,575^ have consolidated information on PROTACs with potential applications in AD. These PROTACs target tau protein, phosphokinase GSK-3β, HDACs, BET proteins, and transthyretin (TTR)-Aβ interaction, exhibiting characteristics such as low dosage requirements, high efficacy, and high target selectivity. As technology continues to advance, PROTACs undergo continuous refinement. For example, the GSK-3 degrader PT-65 (**62**), developed through click chemistry, exhibited a more prolonged effect on p-tau than its GSK-3 warhead (a GSK-3 inhibitor). This may help reduce dosing frequency.^576^ Additionally, phosTAC7 (**63**)^577^ and tau2-8 (**64**)^578^ ingeniously leverage the flexibility of PROTACs to create targeted dephosphorylation strategies. In summary, PROTACs represent a burgeoning technology in AD drug development, specifically targeting dysfunctional enzymes, misfolded proteins, and even PPI in AD through the rational utilization of the UPS clearance system. However, PROTACs are still facing challenges. Limitations include the restricted choices of E3 ligases, primarily CRBN and VHL, and the considerable molecular weight of compounds that cause poor BBB penetration. Notably, while PROTACs can alter the existing pathological phenotype of AD, they cannot reverse the damage that has already occurred, particularly in addressing the genetic mutations associated with FAD (Fig. 6c).^574^

### Targeting the PPI network

Protein-protein interactions (PPIs) are fundamental in maintaining cellular functions, while aberrant interactions between proteins are implicated in the pathogenesis of numerous diseases.^75,579^ For instance, AD is characterized by the misfolding and aggregation of Aβ and tau proteins, involving a variety of molecular mechanisms and complex networks of PPIs.^580–582^ Thus, disrupting these interactions may block some critical signaling pathways and potentially mitigate the pathological process of AD. Although large and flat PPI interfaces may be more conducive to peptide and protein drug targeting,^75,583,584^ small molecule inhibitors also play a role in some AD-related PPIs due to their unique advantages. For example, Aβ can interact with the leukocyte immunoglobulin-like receptor B2 (LilrB2) and negatively mediate synapses and memory.^585^ Compounds ALI6 (**65**)^586^ and **66**^587^ can effectively block this interaction, which reverses the changes in cofilin signaling downstream of LilrB2 and the inhibition of neurite outgrowth, thus protecting neuronal cells from Aβ toxicity. In contrast, the interaction between Aβ and transthyretin (TTR) is a favored PPI, because it reduces Aβ aggregation and toxicity.^588^ Iododiflunisal (**67**, IDIF), luteolin (**68**), and three marketed drugs sulindac (**69**), olsalazine (**70**), and flufenamic (**71**) are small-molecule chaperones for the TTR/Aβ interaction. They all significantly reduced the caspase-3 activation in SH-SY5Y cells, protecting cells from apoptosis/death. Moreover, their good BBB penetration ability warrants their application in TTR target validation and positions them as potential candidates for AD clinical trials.^589^ Kelch-like ECH-associated protein 1 (Keap1)-nuclear factor erythroid 2-related factor 2 (Nrf2), critical for regulating anti-oxidative stress, represents a PPI targetable by covalent inhibitors.^590^ Its orally available inhibitor NXPZ-2 (**72**) effectively ameliorated Aβ-induced cognitive dysfunction in mice by increasing the expression levels of Nrf2 and downstream antioxidant enzymes.^590^ However, issues of low solubility and lack of validation in transgenic AD models with NXPZ-2 (**72**) are presented, which was properly addressed by its analog **73**.^591^ Additionally, another Keap1-Nrf2 PPI inhibitor **74**, which combined conformational features significantly similar to the Keap1-Nrf2 ETGE complex, revealed the unique inhibition mechanism and provided an innovative strategy for the development of new Keap1-Nrf2 PPI inhibitors.^592^ In summary, inhibition or activation of fundamental pathological interactions presents an alternative therapeutic avenue for AD. PPI modulators precisely target pathological pathways in a reversible and mildly regulatory manner, preserving the physiological functions of proteins and thereby reducing severe side effects associated with excessive inhibition, thus offering higher safety levels. In addition, recent advances in computational analysis and model building also support the identification of specific, high-affinity PPI drug hits. These approaches systematically locate underutilized or optimal local interaction regions, simulating the dynamic and transient nature of PPIs, thereby presenting unlimited possibilities for efficient PPI drug discovery (Fig. 7c).^593^

## Conclusions and prospects

AD is a progressive neurodegenerative disease characterized by declining memory and cognitive dysfunction. Pathological features such as Aβ plaques and NFTs in patients have been well documented. However, the existing hypothesis fails to fully elucidate the precise impact of these alterations on the onset and development of AD or the complex interactions among various pathological events. The focus on inflammatory responses and the immune system has led to speculation that certain pathogens such as *Porphyromonas gingivalis*, herpes simplex virus 1 (HSV1), and SARS-CoV-2 may play a role in AD, and the antimicrobial activity of Aβ may also partially supports the mechanism.^214^ Some animal studies suggested that *Porphyromonas gingivalis* could translocate to the brain, closely linked to the deposition of Aβ and tau and the occurrence of neuroinflammation.^594,595^ While some epidemiological data and preclinical studies suggest the association between HSV1 and AD, more research is needed to further validate and understand the relationship.^596–598^ Research of both HSV1-infected mice and AD mouse models has revealed the gene MAM domain containing 2 (MAMDC2) exhibits significant expression in microglia, which results in high levels of I-IFNs to enhance antiviral responses in HSV1-infected mice and neuroinflammation in the AD animal model.^599^ HSV1 may also impact Aβ pathology through mechanisms, such as continuous production and aggregation of Aβ within infected neurons via the activation of caspase 3,^600^ and altering γ-secretase activity.^601^ Many COVID-19 patients diagnosed with some long or post-acute sequelae of COVID-19 such as brain atrophy and memory decline, greatly increasing the risk of AD.^602,603^ AD patients are also more susceptible to COVID-19, with higher risks of hospitalization and mortality in the patients with dementia and COVID-19.^604^ This suggests a correlation between the two diseases. From a genetic perspective, some genes such as APOE4 and oligoadenylate synthetase 1 (OAS1) play important roles in susceptibility to both COVID-19 and AD. APOE4 as a significant genetic risk factor for AD also interacts with angiotensin-converting enzyme 2 (ACE2) to hinder SARS-CoV-2 infection and influence inflammation levels.^605^ Some variants in the interferon-responsive gene OAS1 may lower its expression and potentially increase the likelihood of AD and severe COVID-19, through excessive release of pro-inflammatory signals in myeloid cells such as microglia and macrophages, further leading to cell death.^606^ SARS-CoV-2 affects key pathological changes, such as Aβ, tau, and neuroinflammation, promoting cognitive impairment. Interaction between the SARS-CoV-2 Spike S2 subunit and γ-secretase could regulate γ-secretase cleavage of APP and increase Aβ production.^607^ SARS-CoV-2 may facilitate the intercellular spread of tau aggregates by forming extracellular vesicles modified with spike S protein.^608^ Upon entry into the host cell, it may cause cytokine storms and immune dysregulation, disrupt the BBB, and reduce Aβ clearance, ultimately resulting in neuroinflammation and Aβ aggregation.^602^ Additionally, the upregulation of shared pathogenic kinases in COVID-19 and AD, such as epidermal growth factor receptors, vascular growth factor receptors, Bruton tyrosine kinase, spleen tyrosine kinase, c-ABL, and JAK/STAT, suggests potential interactions between immunological and neurological mechanisms.^609^

The current approaches to addressing AD focus on three main aspects: prevention, early diagnosis, and treatment. Managing modifiable risk factors provides a pathway for AD prevention, which may help reducing cognitive decline and the risk of AD. In early diagnosis, various biomarkers of CSF, blood, urine,^610^ saliva,^611^ and retina,^612^ may contribute to comprehensively reflecting the AD pathological process, serving as potential auxiliary tools that are more convenient, cost-effective, or less invasive. Pharmacotherapy is broadly employed in AD treatment; however, the efficacy or safety of most investigational and clinical drugs is not ideal. Factors such as dose-dependent adverse reactions, the inability to penetrate the BBB and achieve effective therapeutic concentrations, and variations in patient sensitivity and metabolic capacity may all influence outcomes. Here, we elucidate the issue from the perspective of the AD nature and drug development technologies. Firstly, the nature of AD may affect the choice of medication. For instance, the deficiency or mutation in aldehyde dehydrogenase (ALDH2) may influence melatonin administration, which could potentially benefit AD patients experiencing cardiac dysfunction. A study^14^ found that in APP/PS1 mutant mice, the decrease in ALDH2 activity could lead to a cascade of downstream events, including disruption of mitochondrial integrity, accumulation of mitochondrial DNA in the cytoplasm, downregulation of the cGAS-STING-TBK1 signaling pathway, and inhibition of autophagy and mitophagy, ultimately resulting in cardiac disorders. Moreover, the beneficial effects of melatonin on mouse hearts, which depend on the regulation of ALDH2 activity, could not be assessed due to mutations or deficiencies in ALDH2. Secondly, appropriate drug development strategies provide the possibility of safe and effective drugs. These technologies may balance the efficacy and risk through targeting selection (single target/multiple targets, structurally similar targets, undruggable targets, active/non-active sites on targets, protein/PPI), the mode of action on targets (clearance, inhibition, or activation), and the duration and intensity of drug targets. Additionally, the burgeoning development of AI may impact AD due to its advantages in handling complex biomedical big data sets.^613^ AI is currently making preliminary explorations in various aspects of AD, from detection and diagnosis to understanding disease mechanisms, biomarker discovery, clinical trial design, drug discovery, and prognosis prediction. Overall, AI’s integration into various facets of AD research holds promise for advancing our understanding of the disease. ^614–618^
